# Psychometric properties of implementation measures for public health and community settings and mapping of constructs against the Consolidated Framework for Implementation Research: a systematic review

**DOI:** 10.1186/s13012-016-0512-5

**Published:** 2016-11-08

**Authors:** Tara Clinton-McHarg, Sze Lin Yoong, Flora Tzelepis, Tim Regan, Alison Fielding, Eliza Skelton, Melanie Kingsland, Jia Ying Ooi, Luke Wolfenden

**Affiliations:** 1 School of Medicine and Public Health, The University of Newcastle, Callaghan, NSW 2308 Australia; 2 Hunter Medical Research Institute, New Lambton Heights, NSW 2305 Australia; 3 Hunter New England Population Health, Wallsend, NSW 2287 Australia

**Keywords:** Public health, Implementation research, Psychometric, Measure, Factor analysis

## Abstract

**Background:**

Recent reviews have synthesised the psychometric properties of measures developed to examine implementation science constructs in healthcare and mental health settings. However, no reviews have focussed primarily on the properties of measures developed to assess innovations in public health and community settings. This review identified quantitative measures developed in public health and community settings, examined their psychometric properties, and described how the domains of each measure align with the five domains and 37 constructs of the Consolidated Framework for Implementation Research (CFIR).

**Methods:**

MEDLINE, PsycINFO, EMBASE, and CINAHL were searched to identify publications describing the development of measures to assess implementation science constructs in public health and community settings. The psychometric properties of each measure were assessed against recommended criteria for validity (face/content, construct, criterion), reliability (internal consistency, test-retest), responsiveness, acceptability, feasibility, and revalidation and cross-cultural adaptation. Relevant domains were mapped against implementation constructs defined by the CFIR.

**Results:**

Fifty-one measures met the inclusion criteria. The majority of these were developed in schools, universities, or colleges and other workplaces or organisations. Overall, most measures did not adequately assess or report psychometric properties. Forty-six percent of measures using exploratory factor analysis reported >50 % of variance was explained by the final model; none of the measures assessed using confirmatory factor analysis reported root mean square error of approximation (<0.06) or comparative fit index (>0.95). Fifty percent of measures reported Cronbach’s alpha of <0.70 for at least one domain; 6 % adequately assessed test-retest reliability; 16 % of measures adequately assessed criterion validity (i.e. known-groups); 2 % adequately assessed convergent validity (*r* > 0.40). Twenty-five percent of measures reported revalidation or cross-cultural validation. The CFIR constructs most frequently assessed by the included measures were relative advantage, available resources, knowledge and beliefs, complexity, implementation climate, and other personal resources (assessed by more than ten measures). Five CFIR constructs were not addressed by any measure.

**Conclusions:**

This review highlights gaps in the range of implementation constructs that are assessed by existing measures developed for use in public health and community settings. Moreover, measures with robust psychometric properties are lacking. Without rigorous tools, the factors associated with the successful implementation of innovations in these settings will remain unknown

**Electronic supplementary material:**

The online version of this article (doi:10.1186/s13012-016-0512-5) contains supplementary material, which is available to authorized users.

## Background

In the field of implementation science, a considerable number of theories and frameworks are being used to better understand implementation processes and guide the development of strategies to improve the implementation of health innovations [1–3]. Many of these theories and frameworks, however, have not been tested empirically. As such, examining the utility of theories and frameworks has been recognised as critical to advance the field of implementation science [4].

The assessment of implementation theories and frameworks necessitates robust measures of their theoretical constructs. Psychometric properties important for measures of implementation research have been proposed [5] and include the following: reliability (internal consistency and test-retest); validity (construct and criterion); broad application (validated in different settings and cultures); and sensitivity to change (responsiveness). Tools which are acceptable, feasible, and display face and content validity are also particularly useful for researchers in real-world settings [5]. Furthermore, the psychometric characteristics of measures that assess a comprehensive range of implementation constructs have been highlighted as a particular priority area of research [4].

A number of reviews of implementation measures exist [6–13]. Such reviews indicate that the quality of existing measures of implementation constructs is limited. A review by Brennan and colleagues, for example, identified 41 instruments designed to assess factors hypothesised to influence quality improvement in primary care [6]. The review found that while most studies reported the internal consistency of instruments, very few assessed the construct validity of the measures using factor analysis [6]. Similarly, in a review of the psychometric properties of research utilisation measures used in health care, Squires and colleagues found that, of the 97 identified studies (60 unique measures), only 31 reported internal consistency and only 3 reported test-retest reliability [13]. Twenty percent of the included measures had not undergone any type of validity testing, and no studies reported on measure acceptability [13].

There are a number of limitations of previous reviews. Most do not provide comprehensive details of the psychometric properties of included measures [7, 8, 12] or address only a small number of constructs or outcomes relevant to implementation science [8, 10]. Additionally, the majority of these reviews primarily focus on measures developed for use in healthcare settings [6, 9, 11, 13]. Evidence from the field of psychometric research has suggested that, even when administered to similar population groups, changes in measure reliability and validity can occur when a measure developed in one setting is applied to another setting with different characteristics [14, 15].

Currently, a comprehensive review of measures of implementation constructs is being conducted by the Society for Implementation Research Collaboration (SIRC) Instrument Review Project [16, 17]. The SIRC review addresses some of the limitations of past reviews by extracting a range of psychometric properties from identified measures and assessing a more comprehensive range of outcomes [18] and constructs relevant to implementation science [19]. The outcomes of interest in the SIRC review are taken from Proctor and colleagues’ Implementation Outcomes Framework (IOF) and focus on the appropriateness, acceptability, feasibility, adoption, penetration, cost, fidelity, and sustainability of the intervention itself [18]. The constructs of interest for the review are drawn from the Consolidated Framework for Implementation Research (CFIR), which outlines factors or conditions deemed important to support the successful implementation of an intervention [19]. The constructs are grouped under five domains which describe the following: (1) Intervention characteristics (details of the intervention itself); (2) Outer setting (factors of influence which are external to an organisation); (3) Inner setting (internal characteristics of an organisation such as culture and learning climate); (4) Characteristics of individuals (actions and behaviours of individuals within the organisation); and (5) Process (systems and pathways within an organisation) [19].

To date, the SIRC review has uncovered 420 instruments related to 34 of the CFIR constructs and 104 instruments related to Proctor and colleagues’ IOF [16, 17]. At present, the data are available for the measures relevant to the inner setting domain of the CFIR and the IOF [20]. However, while comprehensive, the SIRC review only pertains to measures primarily applied to healthcare or mental health care settings, where the individuals responsible for implementing health-related interventions are most likely to be healthcare professionals [16, 17]. In the field of public health, the implementation of health-related interventions often occurs in non-clinical settings, with non-healthcare professionals responsible for implementing these changes. Therefore, there is a need to identify measures which have been developed specifically to measure constructs important for the implementation of health-related interventions in community settings, where the primary role of the organisations and individuals is not healthcare delivery.

To our knowledge, no previous reviews of measures of implementation constructs have focussed on instruments designed for use in a broad range of community settings. Such measures are of particular interest to public health researchers who are utilising implementation theories or frameworks to support evidence-based practice in these settings. As such, the aim of this study was to (1) systematically review the literature to identify measures of implementation constructs which have been developed in community settings; (2) describe each measure’s psychometric properties; and (3) describe how the domains of each measure align with the five domains and 37 constructs of the CFIR.

## Methods

### Scope of this review

The focus of this review was to identify, from peer-reviewed literature, measures which have been developed for use in community-based (non-clinical settings), and which measure constructs aligned to the CFIR. These measures were then examined to determine their psychometric properties and identify which of the CFIR constructs they captured. In this review, ‘measures’ are defined as surveys, questionnaires, instruments, tools, or scales which contain individual items that are answered or scored using predefined response options. ‘Constructs’ are defined as the broad attributes or characteristics which these items (usually grouped into domains) are attempting to capture. The constructs of interest were chosen to align with the CFIR, as this framework is the most comprehensive and draws together numerous theories which have been developed to guide the planning and evaluation of implementation research and combines them into one uniform theory with overarching domains [19].

### Design

A systematic search and review was conducted to address the broad question of ‘what psychometrically robust measures are currently available to assess implementation research in public health and community settings’. A comprehensive search of peer-reviewed publications was conducted using four electronic databases and the quality of identified measures was assessed using well-established, pre-defined psychometric criteria.

### Eligibility

Publications were included if they (1) were peer-reviewed journal articles reporting original research results; (2) reported research from non-clinical settings; (3) reported details regarding the development of a measure; (4) described a measure which assessed at least one of the 37 CFIR constructs; (5) described a measure which was being applied to a specific innovation or intervention; and (6) used statistical methods to assess the measures’ factor structure.

In this review, clinical settings included the following: hospitals, general practices, allied health facilities such as physiotherapy or dental practices, rehabilitation centres, psychiatric facilities, and any other settings where the delivery of health or mental health care was the primary focus. Non-clinical settings included schools, universities, private businesses, childcare centres, correctional facilities, and any other settings where the delivery of health or mental health care was not the primary focus. Given that an aim of the study was to map the domains of included measures against constructs within the CFIR, it was important that measures displayed a minimum level of construct validity via exploratory or confirmatory factor analysis.

Duplicate abstracts were excluded from the review, as were abstracts describing reviews, editorials, commentaries, protocols, conference abstracts, and dissertations. Publications which reported on measures developed using qualitative methods only were also ineligible.

### Search strategy

A search of MEDLINE, PsycINFO, EMBASE, and CINAHL databases was conducted to identify publications describing the development of measures to assess factors relevant to the implementation of innovations. These four databases were selected as they index journals from the field of implementation science and provide extensive coverage of research across a range of public health and community settings, such as schools, pharmacies, businesses, nursing homes, sporting clubs, and childcare facilities.

Prior to the database searches being conducted, four authors met to ensure that the chosen keywords accurately captured the constructs of interest and that keywords were combined using the correct Boolean operators [21]. The core search terms comprised of keywords that related to measurement, the psychometric properties of instruments, the levels at which the measurement could occur (e.g. organisational or individual) and the goals of research implementation. These keywords were as follows: [questionnaire or measure or scale or tool] AND [psychometric or reliability or validity or acceptability] AND [organisation* or institut* or service or staff or personnel] AND [implement* or change or adopt* or sustain*].

Similar to the strategy used in the SIRC review [16, 17], the core search terms were combined with five more keyword searches designed to capture the constructs within each of the five CFIR domains: (1) Intervention Characteristics [strength or quality or advantage or adapt* or complex* or pack* or cost]; (2) Outer Setting [needs or barrier* or facilitate* or resource* or network or external or peer or compet* or poli* or regulation* or guideline* or incentive*]; (3) Inner Setting [structur* or communication or cultur* or value* or climate or tension or risk* or reward* or goal* or feedback or commitment or leadership or knowledge*]; (4) Characteristics of Individuals [belief* or attitude* or self-efficacy or skill* or identi* or trait* or ability* or motivat*]; or (5) Process [plan* or market or train or manager or team or champion or execut* or evaluat*].

The keyword search terms were repeated for all four databases. Keyword searches were limited to the English language; however, no limit was placed on the year of publication, as measurement tools often evolve over many years. Medical Subject Headings (MeSH) were not used in the literature search, as keyword searches have been found to have higher sensitivity, being more successful than subject searching in identifying relevant publications [22].

### Identification of eligible publications

One author coded all abstracts according to the inclusion and exclusion criteria. A second author cross-checked 10 % of the abstracts to confirm they had been correctly classified. Full-text versions of publications were obtained for included abstracts. To ensure that no relevant tools had been missed, previous systematic reviews [7, 8, 10] were also screened for relevant measures, as were tools included on the SIRC Instrument Review Project website [20]. Copies of publications for any additional measures that met the inclusion criteria were obtained. Full-text versions of all eligible publications were then obtained and screened to identify the names and acronyms of all relevant measures they described. The reference lists of all eligible publications were also screened for any additional measures, and Google Scholar was used to conduct cited reference searches. A final literature search was conducted by ‘measure name’ and ‘author names’, using Google Scholar. This search strategy ensured that as many publications as possible were found that related to the psychometric development and validation and revalidation and cross-cultural adaptation of identified measures.

### Extraction of data from eligible publications

The properties of each measure were extracted from all full-text publications relating to the development of the measure using data reported in the manuscript text, tables, or figures. Extracted data included: (1) the research setting, sample, and characteristics of the intervention or innovation being assessed; (2) psychometric properties including face and content validity, construct and criterion validity, internal consistency, test-retest reliability, responsiveness, acceptability, and feasibility; and (3) whether the measure had undergone a process of revalidation or cross-cultural adaptation.

The psychometric properties of each measure were independently assessed by two authors using the same criteria described in previous systematic reviews [23, 24] and according to the guidelines for the development and use of tests, including the *Standards for Educational and Psychological Testing* [5, 25, 26]. The *Standards* provides a frame of reference to ensure all relevant issues are addressed when developing a measure and allows the quality of measures to be evaluated by those who wish to use them [25]. Following the assessment of psychometric properties, two authors then independently coded each publication to determine which measure domains corresponded with which CFIR constructs. When discrepancies emerged, a third author assisted in reaching consensus.

### Psychometric coding

#### Setting, sample, and characteristics of the innovation being assessed

Details regarding the country and setting where the measure was developed, characteristics of the innovation or intervention being assessed, response rate, sample size, and demographic characteristics of the sample (gender and profession) who completed the measure were described.

#### Face and content validity

An instrument is said to have face validity if both the administrators and those who complete it agree that it measures what it was designed to measure [27]. To have content validity, the description of the measure’s development needed to include: (1) the process by which items were selected; (2) who assessed the measure’s content; and (3) what aspects of the measure were revised [14, 28]. Information regarding any theories or frameworks that the measure was developed to test, as well as whether items were adapted from previously validated measures, was also extracted.

#### Construct and criterion validity

A measure was classified as having good internal structure (construct validity) if exploratory factor analysis (EFA) was performed with eigenvalues set at >1 [14, 29] and >50 % of the variance was explained [30], or confirmatory factor analysis (CFA) was performed with a root mean square error of approximation (RMSEA) of <0.06 and a comparative fit index (CFI) of >0.95 [31, 32]. The number of items and domains in the measure following factor analysis was recorded. Additional construct validity was determined by assessing whether the measure had convergent validity (correlations (*r*) >0.40) with similar instruments or divergent validity (correlations (*r*) <0.30) with dissimilar instruments [33]. Criterion validity was determined by assessing whether the measure was able to obtain different scores for sub-populations with known differences (known-groups validity) [34].

#### Internal consistency and test-retest reliability

To meet the criteria for internal consistency, correlations for a measure’s subscales and total scale needed to have a Cronbach’s alpha (*α*) of >0.70 or a Kuder-Richardson 20 (KR-20) of >0.70 for dichotomous response scales [28]. For test-retest reliability, the measure needed to have undergone a repeated administration with the same sample within 2–14 days [35]. Agreement between scores from the two administrations needed to be calculated, with item, subscale, and total scale correlations having a (1) Cohen’s kappa coefficient (*κ*) of >0.60 for nominal or ordinal response scales [14]; (2) Pearson correlation coefficient (*r*) of >0.70 for interval response scales [14, 28]; or an (3) Intraclass correlation coefficient (ICC) of >0.70 for interval response scales [14, 28].

#### Responsiveness, acceptability, feasibility, revalidation, and cross-cultural adaptation

A measure’s potential to detect change over time was confirmed if it could show a moderate effect size (>0.5) for a given change [14, 28, 36], and if it had minimal floor and ceiling effects (less than 5 % of the sample achieved the highest or lowest scores) [37]. To determine acceptability and feasibility (burden associated with using the measure), data on the following were extracted: proportion of missing items, time needed to complete, and time needed to interpret and score [28]. Data from publications reporting the revalidation of a measure with additional samples, or in different languages or cultures, were also extracted [28].

### CFIR coding

The domains of each included measure were assessed to determine whether the factors they measured corresponded with one or more of the 37 CFIR constructs [19]. A brief summary of each of the CFIR constructs is presented in Additional file 1. The mapping process was domain-focused (i.e. mapping the overall measure domains to constructs) rather than item-focused (i.e. mapping individual items to constructs) to ensure that the overall construct was well captured. Within a measure, only one domain needed to be judged by the reviewers to address a CFIR construct. Therefore, it was possible that a measure with five domains might only have one of its domains mapped to a CFIR construct. Similarly, a measure with three domains might have all contributing to the same CFIR construct. In the latter scenario, the construct was only counted once.

### Analysis

Descriptive statistics (frequencies and proportions) were used to report the number of domains from the included measures which were mapped to each of the CFIR constructs and CFIR domains. Frequencies and proportions were also used to describe the number of measures which met various psychometric criteria.

## Results

### Identified measures of implementation constructs

**Fig. 1:**
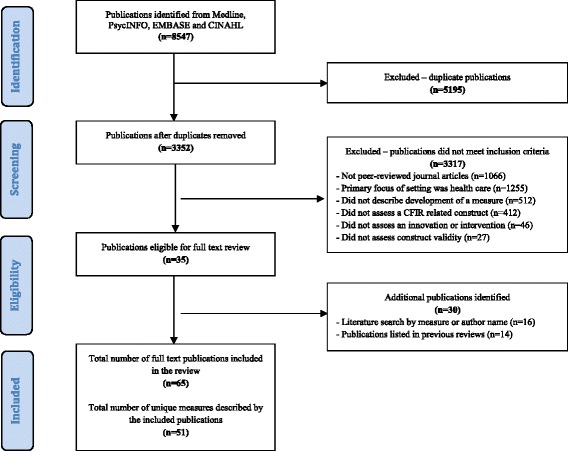
PRISMA flow diagram of the publication and measure inclusion process

The initial searches of MEDLINE, PsycINFO, EMBASE, and CINAHL identified 8547 potentially relevant publications. Of these, 5195 were duplicates leaving 3352 publication abstracts to be coded. Of these 3352 publications, 3317 did not meet the inclusion criteria (see Fig. 1 for PRISMA diagram), leaving 35 eligible publications. The process of identifying measures included in systematic reviews related to the current review [7, 8, 10], and a secondary literature search by measure or author name, lead to the inclusion of an additional 30 publications. A total of 65 full-text publications were retained which described 51 unique measures.

### Psychometric properties of measures

#### Setting, sample, and characteristics of the innovation being assessed

Table 1 outlines the details of the setting, sample, and characteristics of the innovation being assessed by each measure. The majority of measures were developed in the USA (*n* = 28), with Canada and Australia also having developed three or more measures each. Sixteen measures were developed for use in school settings [38–52], six for use in universities or colleges [53–60], three for use in pharmacies [61–63], two for use in police or correctional facilities [64, 65], two for use in nursing homes [66, 67], six for use with whole communities or in multiple settings [68–75], and sixteen measures were developed for use in workplace settings or other organisations (e.g. utility companies, IT service providers, human services) [76–92]. A broad range of innovations or interventions were assessed, with technology-focussed innovations featuring prominently. Sample sizes in each study ranged from 31 to 1358, and response rates ranged from 15 to 98 %. Sample characteristics (i.e. gender and profession of participants) were inconsistently reported across the studies.

**Table 1:** Setting, sample, and characteristics of the innovation being assessed

Measure	Country	Setting	Characteristics of the innovation being assessed	Sample size	Response rate	Gender and profession of participants (Standard 1.8)^a^
Schools						
Adopter Characteristics Scale [43]	USA	Schools in Texas (number not reported)	Adoption and use of a classroom educational programme for tobacco prevention	*n* = 131	54 %	Second grade teachers
Awareness and Concern Instrument [51]	USA	School districts in North Carolina with at least two junior high or middle schools (*n* = 21)	Awareness, concern, and interest in adopting and implementing tobacco prevention the curricula	*n* = 917	Not reported	Central office administrators Principals Teachers
Health Teaching Self-Efficacy (HTSE) Scale [47]	USA	Schools in the Region IV district of the Texas Education Agency (number not reported)	Ability to implement health teaching in the classroom	*n* = 31	Not reported	Junior high teachers Senior high teachers
Index of Inter-professional Team Collaboration-Expanded School Mental Health (IITC-ESMH) [50]	USA	Schools across the USA (number not reported)	Level of inter-professional collaboration occurring to implement learning support and mental health promotion strategies in schools	*n* = 436	Not reported	Female = 88 % School-employed mental health workers (e.g. school counsellors) Community-based mental health workers (e.g. clinical psychologists) School nurses Educators
McKinney-Vento Act Implementation Scale (MVAIS) [40]	USA	Illinois Association of School Social Workers annual conference	Perceived knowledge and awareness regarding implementation of the McKinney-Vento Act	*n* = 201	40 %	Female = 92 % School social workers
Organisational Climate Instrument [51]	USA	School districts in North Carolina with at least two junior high or middle schools (*n* = 21)	Perceptions regarding the organisational climate in schools adopting tobacco prevention curricula	*n* = 910	Not reported	Central office administrators Principals Teachers
Perceived Attributes of the Healthy Schools Approach Scale [42]	Canada	Schools in Quebec (*n* = 107)	Perceived attributes of a health promoting school initiative	*n* = 141	28 %	Female = 62 % Principals School health promotion delegates
Policy Characteristics Scale [52]	Belgium	Flemish secondary schools, including community schools, and subsidised public and private schools (*n* = 37)	Perceptions of a new teacher evaluation policy	*n* = 347	82 %	Female = 58 % Teachers
Role-Efficacy Belief Instrument (REBI) [45]	USA	Schools in a state recently mandating sexuality education (number not reported)	Role-efficacy related to the successful implementation of a mandated sexuality curriculum	*n* = 123	Not reported	Female = 67 % County-level curriculum coordinators
Rogers’s Adoption Questionnaire [51]	USA	School districts in North Carolina with at least two junior high or middle schools (*n* = 21)	Perceptions regarding the characteristics of three different tobacco prevention curricula	*n* = 251	Not reported	Central office administrators Teachers
School Wellness Policy Instrument (WPI) [48]	USA	Public elementary schools in Mississippi (*n* = 30)	Acceptance and implementation of nutrition competencies as part of a school wellness policy	*n* = 947	34 %	Teachers
School-level Environment Questionnaire—South Africa (SLEQ-SA) [38]	South Africa	Secondary schools in the Limpopo Province (*n* = 54)	Perceptions of the school-level environment with regard to the implementation of outcomes-based education	*n* = 403	Not reported	Teachers
School Success Profile—Learning Organisation (SSP-LO) Measure [39]	USA	Public middle schools in North Carolina (*n* = 11)	Perceptions of organisational learning as part of an evaluation of the effectiveness and sustainability of the School Success Profile Intervention Package	*n* = 766	80 %	Teachers Specialists Teacher assistants Administrators Other employees (e.g. cafeteria workers)
School Readiness for Reforms—Leader Questionnaire (SRR-LQ) [41]	USA	Elementary, middle, and high schools in nine districts from South-west Florida (*n* = 169)	Perceptions regarding school readiness for the implementation of reforms including standards-based testing	*n* = 167	99 %	Leaders from elementary schools (e.g. principals, assistant principals, curriculum specialists) Leaders from high schools Leaders from middle schools
School-wide Universal Behaviour Sustainability Index—School Teams (SUBSIST) [46, 49]	USA	Elementary, middle, and secondary public schools in Maryland, Michigan, Minnesota, New Hampshire, and Oregon who were implementing school-wide positive behaviour support (*n* = 14)	Evaluation of school capacity to sustain school-wide positive behaviour support	*n* = 25	Not reported	Internal school team leaders External district coaches
Teacher Receptivity Measure [44]	USA	Elementary schools in Texas (number not reported)	Views towards the implementation of the smoke-free class of 2000 teaching kit	*n* = 216	79 %	First grade teachers
Universities/colleges						
Intention to Adopt Mobile Commerce Questionnaire [54, 55]	Kazakhstan	Private institutions of higher learning in Almaty and Astana (*n* = 3)	Intention to adopt mobile commerce	*n* = 345	51 %	Female = 50 % University students
Perceived Attributes of eHealth Innovations Questionnaire [53]	USA	Education providers including community colleges, state colleges and universities from across the United States (*n* = 12)	Perceived attributes of a technological innovation for health education	*n* = 193	84 %	Male = 53 % Sophomores Freshman Juniors Seniors
Perceived Usefulness and Ease of Use Scale [56]	USA	Business school at Boston University	Evaluation of usefulness and ease of use of two computer programmes	*n* = 40	93 %	MBA students
Post-adoption Information Systems Usage Measure [59]	USA	University in the United States	Post-adoption perceptions of a self-service, web-based student information system	*n* = 1008	Not reported	Females = 53 % University students
Social Influence on Innovation Adoption Scale [60]	Australia	University of South Australia	Attitudes towards adopting advanced features of email software	*n* = 275	15 %	Female = 64 % Academic staff Professional staff
Tertiary Students Readiness for Online Learning (TSROL) Scale [57, 58]	Australia	Metropolitan university in Australia	Readiness to adopt an online approach to teaching and learning	*n* = 254	52 %	Female = 65 % University students
Pharmacies						
Facilitators of Practice Change Scale [63]	Australia	Australian community pharmacies (*n* = 735)	Facilitators of practice change with regard to the implementation of cognitive pharmaceutical services in community pharmacies	*n* = 1303	Not reported	Proprietor pharmacists Employee pharmacists Pharmacy assistants (including technicians)
Leeds Attitude Towards Concordance (LATCon) Scale [62]	Finland	Finnish community pharmacies (number not reported)	Attitudes towards the implementation of a new counselling model based on concordance and mutual decision-making between pharmacists and patients	*n* = 376	51 %	Community pharmacists
Perceived Barriers to the Provision of Pharmaceutical Care Questionnaire [61]	China	Community pharmacies in Xian, China (number not reported)	Attitudes and barriers to the implementation of pharmaceutical care	*n* = 101	78 %	Female = 82 % Prescription checking staff Quality assurance staff Staff managers
Police/correctional facilities						
Perceptions of Organisational Readiness for Change [65]	USA	Juvenile justice offices (*n* = 12)	Perceptions of organisational readiness to implement an innovation consisting of screening, assessment, and referral strategies	*n* = 231	Not reported	Female = 58 % Case-workers Managers Front-line supervisors
Receptivity to Organisational Change Questionnaire [64]	USA	Districts I and II of the Hillsborough County, Florida Sheriff’s Office (*n* = 2)	Attitudes regarding an agency-wide shift towards community-oriented policing	*n* = 204	98 %	Patrol deputies Other sworn employees
Nursing homes						
Intervention Process Measure (IPM) [67]	Denmark	Elderly care centres (*n* = 2)	Assessment of employee perceptions related to the implementation of self-managed teams	*n* = 163	81 %	Female = 93 % Healthcare assistants Nurses Other health educated staff Staff with no healthcare education
Staff Attitudes to Nutritional Nursing (SANN) Care Scale [66]	Sweden	Residential units in a municipality of southern Sweden (*n* = 8)	Attitudes of nursing staff towards the implementation of nutritional nursing care	*n* = 176	95 %	Registered nurses Nurse aids
Whole communities/multiple settings						
4-E Telemeter [70, 71]	Netherlands	Education-related settings from 39 countries including elementary, secondary, university, vocational, and company training settings (number not reported)	Likelihood of using telecommunications-related technological innovations in learning-related settings	*n* = 550	Not reported	Instructors Students Administrators Educational support unit members Technical support unit members Researchers
Attitudes Towards Asthma Care Mobile Service Adoption Scale [94]	Taiwan	General community	Attitudes and behavioura towards the adoption of a asthma care mobile service	*n* = 229	57 %	Male = 63 %
Intention to Adopt Multimedia Messaging Service Scale [69]	Taiwan	General community	Attitudes towards and intention to adopt multimedia messaging services	*n* = 112	Not reported	Male = 55 % Students Electronics or IT sector employees Education sector employees Financial services, entertainment, media, government, health care, and law office employees
Systems of Care Implementation Survey (SOCIS) [68, 72]	USA	Education settings, mental health services, family assistance organisations, child welfare services, juvenile justice services and medical services from 225 counties across the USA (number not reported)	Level of implementation of factors contributing to effective children’s systems of care	*n* = 910	42 %	Female = 72 %
Stages of Concern Questionnaire (SoCQ) [73, 74]	USA	Elementary schools and higher education institutions in the USA (number not reported)	Concerns about innovations including team teaching in elementary schools and using instructional modules in colleges	*n* = 830	Not reported	Public school teachers College professors Specialists Teacher assistants Administrators Other employees (e.g. cafeteria workers)
Telepsychotherapy Acceptance Questionnaire [75]	Canada	First nations communities in Quebec (*n* = 32)	Attitudes and perceptions of adoption and referral to telepsychotherapy delivered via videoconference	*n* = 205	77 %	Female = 70 % Community elders/natural helpers Social assistants/social workers Nurses/school nurses Psychoeducators Educators/teachers Psychologists Social interveners Community health representatives Non-professionals
Other workplaces/organisations						
Adoption of Customer Relationship Management Technology Scale [88]	USA	A national buying cooperative for hardware and variety businesses in the USA	Facilitators to adopting customer relationship management technology	*n* = 386	48 %	Owner-operators of hardware and variety businesses
Coping with Organisational Change Scale [83]	USA	International organisations including Australian banks, a Scandinavian shipping company, a United Kingdom oil company, a US university and a Korean manufacturing company (*n* = 6)	Ability to cope with organisational change including reorganisation and downsizing, managerial changes, mergers and acquisitions, and business divestments	*n* = 514	71 %	Male = 91 % Middle and upper-level managers
Data Mining Readiness Index (DMRI) [80]	Malaysia	Telecommunication organisations (number not reported)	Readiness to adopt data mining technologies	*n* = 106	43 %	Telecommunications employees
Group Innovation Inventory (GII) [78, 91]	USA	High-technology companies, primarily in the aerospace and electronics industries (number not reported)	Attitudes towards innovation within groups developing new component-testing programmes, systems-level integration projects, engineering audit procedures, and failure analyses	*n* = 244	Not reported	Managers/supervisors Engineers/scientists
Intention to Adopt Electronic Data Interchange Questionnaire [79]	Canada	Purchasing Managers’ Association of Canada (PMAC)	Intention to adopt electronic data interchange	*n* = 337	58 %	Senior purchasing managers
Organisational Change Questionnaire-Climate of Change, Processes, and Readiness (OCQ-C, P, R) [77]	Belgium	Belgian organisations from sectors including information technology, petrochemicals, telecommunications, consumer products, finance, insurance, consultancy, healthcare, and government services (n = 42)	Attitudes towards recently announced, large-scale change including downsizing, reengineering, total quality management, culture change, and technological innovation	n = 1358	Not reported	Male = 64 %
Organisational Learning Capacity Scale (OLCS) [76]	USA	Human service organisations from a Southern United States city (*n* = 5)	Organisational readiness for change towards primary prevention, strength-based approach, empowerment, and changing community conditions	*n* = 125	50 %	Female = 79 %
Organisational Capacity Measure-Chronic Disease Prevention and Healthy Lifestyle Promotion [81]	Canada	Public health organisations in Canada including government departments, regional health authorities, public health units, and other professional and non-government organisations (*n* = 216)	Organisational capacity in public health systems to develop, adopt, or implement chronic disease prevention and healthy lifestyle programmes	*n* = 216	Not reported	Senior/middle managers Service providers Professional staff
Organisational Environment and Processes Scale [89]	USA	Fortune 1000 companies (manufacturing firms, service organisations) and large government agencies (*n* = 710)	Perceptions of structures and processes related to the adoption of an administrative innovation (Total Quality Management) by Information Systems departments in organisations	*n* = 123	17 %	Senior Information Systems Executives
Perceived Characteristics of Innovating (PCI) Scale [87]	Canada	Utility companies, resource-based companies, government departments and a natural grains pool (*n* = 7)	Perceptions regarding the adoption of personal work stations	*n* = 540	68 %	Executive and middle management First-line supervisors Non-management professionals Technical and clerical staff
Perceived Strategic Value and Adoption of eCommerce Scale [90]	Ghana	Small and medium-sized enterprises (200 employees or less) (*n* = 107)	Perceived strategic value of adopting eCommerce	*n* = 107	54 %	Owners/managers
Perceived eReadiness Model (PERM) Questionnaire [85, 86]	South Africa	Business organisations in South Africa (*n* = 875)	Readiness to adopt eCommerce	*n* = 150	19 %	Chief Executive Officers Managing directors General managers Directors of finance, IT, eCommerce and marketing
Readiness for Organisational Change Measure [82]	USA	Government organisation responsible for developing fielding information systems for the Department of Defence	Readiness for a new organisation structure that clarified lines of authority and eliminated duplicate functions	*n* = 264	53 %	Male = 59 % Computer analysts and programmers
Technology Acceptance Model 2 (TAM2) Scale [96]	United States	Manufacturing firm, financial services firm, accounting services firm, international investment banking firm (n = 4)	Perceived usefulness and ease of use of a new software system	n = 156	78 %	Manufacturing floor supervisors Financial services workers Accounting services workers Investment banking workers
Total Quality Management (TQM) and Culture Survey [92]	USA	Manufacturing firm, non-profit service agency, university (*n* = 3)	Perceived organisational culture and implementation of total quality management practices	*n* = 886	95 %	Manufacturing firm employees Non-profit service agency employees Government employees Service agency employees Educational institute employees
Worksite Health Promotion Capacity Instrument (WHPCI) [84]	Germany	Information and communication technology companies in Germany (*n* = 522)	Willingness and capacity to implement worksite health promotion activities	*n* = 522	21 %	Managing director/board of directors Division head/senior department head Department head Human resources manager/director Owner/proprietor Assistant to executive management

^a^ Standard 1.8—Describe the composition of the sample from which validity evidence is obtained, including relevant sociodemographic characteristics [25]

#### Face and content validity

Almost all measures (*n* = 47) had undergone a process of face and content validation. The development of 36 measures was guided by an existing theory or framework (Additional file 2). No measures were specifically designed to address all constructs considered important for the implementation of innovations by the CFIR. Twenty-six measures had adapted at least some of their items from pre-existing instruments (Additional file 2).

#### Construct and criterion validity

The internal structure of 45 instruments was determined via EFA (11 of these also used CFA [42, 49, 52, 54, 55, 59, 65, 67, 77, 78, 82, 91–93]), and six studies used CFA alone [39, 40, 68, 72, 75, 83, 94] (Additional file 3). For studies which conducted EFA, 46 % reported that >50 % of the variance was explained by the final factor model. None of the studies that used CFA alone reported acceptable RMSEA (<0.06) or CFI (>0.95). Across all measures, the number of items ranged from 9 to 149, and the number of factors (domains) ranged from 1 to 20. Eight measures were tested for criterion validity for sub-populations with known differences. These measures demonstrated capacity to distinguish between a number of groups with known differences, including the amount of teaching experience [47], familiarisation with technology [59], age [58], and managers and non-managers [77]. Only two measures [41, 82] reported testing for convergent/divergent validity against existing instruments, although only one [82] met the required threshold of having significant positive or negative correlations >0.40 or <0.30 with an external measure. In this instance, these relationships were only reported for some individual domains rather than the total score of the scale.

#### Internal consistency and test-retest reliability

Fifty of the 51 included measures reported on the internal consistency of either the total scale or the individual domains (Additional file 4). The internal consistency of both the total scale and the domains was reported for four measures [40, 61, 66, 76], the internal consistency of the total scale only was reported for five measures (all alpha’s >0.70) [47, 49, 51, 75, 83], and the internal consistency for the scale domains only was reported for the remaining 41 measures. Twenty measures achieved a Cronbach’s alpha of >0.70 for all of their domains [38, 40, 41, 48, 50–52, 54, 59, 60, 63, 76, 79, 81, 84, 85, 87, 89, 90, 95, 96], indicating that more than 50 % of measures did not meet the acceptable threshold for at least one domain. Three measures were examined for test-retest reliability [47, 73, 84]. The administration period was acceptable (2–14 days) for all measures, and adequate test-retest reliability (Pearson’s correlations >0.70) was achieved for all measures, with the exception of one domain (awareness, *r* = 0.65) in the Stages of Concern Questionnaire [74].

#### Responsiveness, acceptability, feasibility, revalidation, and cross-cultural adaptation

Seventeen measures reported acceptability and feasibility, with five studies reporting the time that it took to complete the measure (range 10–70 min; *M =* 34.6 min) [39, 64, 73, 81, 90] and six studies reporting the proportion of missing items observed following the measure administration (range 1.5–5 %) [52, 59, 63, 67, 75, 84] (Additional file 5). Seven studies examined responsiveness in relation to effect sizes [38, 47, 67, 69, 75, 93, 97], and all but one reported an effect size above the threshold criterion of 0.5 [67], indicating that these measures are capable of detecting moderate size change (Additional file 5). No studies reported floor or ceiling effects. Thirteen measures were revalidated in new settings and with different populations across a number of additional studies [55, 77, 91, 96, 98–112].

A summary of the psychometric criteria reported by the included measures can be seen in Table 2.

**Table 2:** Summary of psychometric properties reported for each measure

Measure	Face/content validity	Construct validity	Criterion validity	Internal consistency	Test-retest reliability	Responsiveness	Acceptability/feasibility	Revalidation/cross-cultural
Schools								
Adopter Characteristics Scale [43]	–	✓	–	✓	–	–	–	–
Awareness and Concern Instrument [51]	✓	✓	–	✓	–	–	–	–
Health Teaching Self-efficacy (HTSE) Scale [47]	✓	✓	✓	✓	✓	✓	–	–
Index of Inter-professional Team Collaboration-Expanded School Mental Health (IITC-ESMH) [50]	✓	✓	–	✓	–	–	–	–
McKinney-Vento Act Implementation Scale (MVAIS) [40]	✓	✓	–	✓	–	–	–	✓
Organisational Climate Instrument [51]	✓	✓	–	✓	–	–	–	–
Perceived Attributes of the Healthy Schools Approach Scale [42]	✓	✓	–	✓	–	–	–	–
Policy Characteristics Scale [52]	✓	✓	–	✓	–	–	✓	–
Role-Efficacy Belief Instrument (REBI) [45]	✓	✓	–	✓	–	–	–	–
Rogers’s Adoption Questionnaire [51]	✓	✓	–	✓	–	–	–	–
School Wellness Policy Instrument (WPI) [48]	✓	✓	–	✓	–	–	–	–
School-level Environment Questionnaire—South Africa (SLEQ-SA) [38]	✓	✓	✓	✓	–	✓	–	✓
School Success Profile-Learning Organisation Measure (SSP-LO) [39]	✓	✓	–	✓	–	–	✓	✓
School Readiness for Reforms-Leader Questionnaire (SRR-LQ) [41]	✓	✓	–	✓	–	–	–	–
School-wide Universal Behaviour Sustainability \Index-School Teams (SUBSIST) [49]	✓	✓	–	✓	✓	–	–	–
Teacher Receptivity Measure [44]	✓	✓	–	✓	–	–	–	–
Universities/colleges								
Intention to Adopt Mobile Commerce Questionnaire [54, 55]	✓	✓	–	✓	–	–	–	✓
Perceived Attributes of eHealth Innovations Questionnaire [53]	✓	✓	–	✓	–	–	–	–
Perceived Usefulness and Ease of Use Scale [56]	✓	✓	–	✓	–	–	–	✓
Post-adoption Information Systems Usage Measure [59]	✓	✓	✓	✓	–	–	✓	–
Social Influence on Innovation Adoption Scale [60]	✓	✓	–	✓	–	–	–	–
Tertiary Students Readiness for Online Learning (TSROL) Scale [57, 58]	✓	✓	✓	✓	–	–	–	–
Pharmacies								
Facilitators of Practice Change Scale [63]	✓	✓	–	✓	–	–	✓	–
Leeds Attitude Towards Concordance (LATCon) Scale (Pharmacists) [62]	✓	✓	–	✓	–	–	–	✓
Perceived Barriers to the Provision of Pharmaceutical Care Questionnaire [61]	✓	✓	–	✓	–	–	–	–
Police/correctional facilities								
Perceptions of Organisational Readiness for Change [65]	–	✓	–	✓	–	✓	–	–
Receptivity to Organisational Change Questionnaire [64]	–	✓	–	✓	–	–	✓	–
Nursing homes								
Intervention Process Measure (IPM) [67]	✓	✓	–	✓	–	✓	✓	–
Staff Attitudes to Nutritional Nursing Care (SANN) Scale [66]	✓	✓	–	✓	–	–	–	✓
Whole communities/multiple settings								
4-E Telemeter [70, 71]	✓	✓	✓	–	–	–	–	–
Attitudes Towards Asthma Care Mobile Service Adoption Scale [94]	✓	✓	–	✓	–	✓	✓	–
Intention to Adopt Multimedia Messaging Service Scale [69]	✓	✓	–	✓	–	✓	✓	–
Systems of Care Implementation Survey (SOCIS) [68, 72]	✓	✓	✓	✓	–	–	–	–
Stages of Concern Questionnaire (SoCQ) [73, 74]	✓	✓	–	✓	✓	–	✓	✓
Telepsychotherapy Acceptance Questionnaire [75]	✓	✓	–	✓	–	✓	–	–
Other workplaces/organisations								
Adoption of Customer Relationship Management Technology Scale [88]	✓	✓	–	✓	–	–	–	–
Coping with Organisational Change Scale [83]	✓	✓	–	✓	–	–	–	–
Data Mining Readiness Index (DMRI) [80]	✓	✓	–	✓	–	–	–	–
Group Innovation Inventory (GII) [78, 91]	✓	✓	–	✓	–	–	–	✓
Intention to Adopt Electronic Data Interchange Questionnaire [79]	✓	✓	–	✓	–	–	–	–
Organisational Change Questionnaire-Climate of Change, Processes, and Readiness (OCQ-C, P, R) [77]	✓	✓	✓	✓	–	–	–	✓
Organisational Learning Capacity Scale (OLCS) [76]	✓	✓	–	✓	–	–	–	–
Organisational Capacity Measure-Chronic Disease Prevention and Healthy Lifestyle Promotion [81]	✓	✓	–	✓	–	–	–	–
Organisational Environment and Processes Scale [89]	–	✓	–	✓	–	–	–	–
Perceived Characteristics of Innovating (PCI) Scale [87]	✓	✓	✓	✓	–	–	–	✓
Perceived Strategic Value and Adoption of eCommerce Scale [90]	✓	✓	–	✓	–	–	–	–
Perceived eReadiness Model (PERM) Questionnaire [85, 86]	✓	✓	–	✓	–	✓	–	✓
Readiness for Organisational Change Measure [82]	✓	✓	–	✓	–	–	–	–
Technology Acceptance Model 2 (TAM2) Scale [96]	✓	✓	–	✓	–	–	–	✓
Total Quality Management (TQM) and Culture Survey [92]	✓	✓	–	✓	–	–	–	–
Worksite Health Promotion Capacity Instrument (WHPCI) [84]	✓	✓	–	✓	–	–	✓	–

#### Mapping of measure domains that align with the 37 constructs of the CFIR

The number of measure domains that mapped onto the CFIR constructs ranged from 1 to 19. Relative advantage, networks and communications, culture, implementation climate, learning climate, readiness for implementation, available resources, and reflecting and evaluating were the constructs most frequently addressed by the included measures. Five of the CFIR constructs were not addressed by any measure (Additional file 6). These five constructs were as follows: intervention source, tension for change, engaging, opinion leaders, and champions.

## Discussion

To our knowledge, this is the first systematic review to describe the psychometric properties of measures developed to assess innovations and implementation constructs specifically in public health and community settings. Overall, the psychometric properties of included measures were typically inadequately assessed or not reported. No single measure reported on all key psychometric quality indicators. The majority of studies assessed face, content, construct validity, and internal consistency. However, criterion validity (known-groups), test-retest reliability, and acceptability and feasibility were rarely reported. Only seven measures had responsiveness to change assessed. These findings mirror those of previous reviews [7, 13] that found that few measures demonstrated test-retest reliability, acceptability, or criterion validity.

When measures did report psychometric data, it was typically below the widely accepted thresholds defined in this review. Almost half of the measures that reported undertaking EFA reported that their final factor model explained <50 % of the variance. Furthermore, none of the measures that used CFA alone reported satisfactory RMSEA (<.06) or CFI (>0.95). This suggests that a notable proportion of available implementation measures developed and currently available for use in non-clinical settings are not particularly robust or are prone to misspecification of fit. That only eight of the 51 measures explored criterion validity using known-groups is also concerning. The lack of attention to known-groups validity limits the confidence we can place in these measures being able to detect how groups within community settings (e.g. experienced teachers vs. new teachers) vary in regards to implementation of innovation. This is important for identifying which aspects of an intervention or innovation might need to be adjusted to ensure more robust implementation in the future.

Internal consistency was frequently reported but only 40 % of measures reported that all scale domains had a Cronbach’s alpha >0.70, highlighting a need for further refinement of scale items and revalidation. Only three measures assessed test-retest reliability, another area requiring much greater attention in future studies. Those studies that did assess test-retest reliability performed well, meeting the vast majority threshold criteria. However, the stability of these types of measures over time remains unclear. Acceptability and feasibility data were reported for just 33 % of the measures. Mean completion time for measures was almost 35 min. Although shorter questionnaires have been shown to improve response rates [113], it is unclear what the optimal survey length is while still maintaining the survey validity. Rates of missing data ranged from 1.5 to <5 %, which according to Schafer [114] is acceptable given missing data rates of less than 5 % are likely to be inconsequential. Only 25 % of measures had been revalidated or validated in a different culture. This limits the generalisability of the measures and poses a significant barrier to research translation within potentially underserved communities or cultures [115].

Without more comprehensive assessment of the psychometric properties of these instruments, the ability to ascertain the utility of theories or frameworks to support the implementation of innovations in public health and community settings is limited. For example, understanding the responsiveness of measures is essential for evaluating implementation interventions and ensuring that changes in constructs over time can be detected [116, 117]. Having measures which are acceptable and feasible is also important to the conduct of rigorous research, particularly in more pragmatic research studies [5, 18]. Low survey response rates or high rates of attrition due to onerous research methods can introduce bias and compromise study internal and external validity [118, 119].

### Alignment of measure domains with constructs of the CFIR

While some of the CFIR constructs were addressed by domains from multiple measures in this study, five constructs were not assessed by any measure. These were intervention source, tension for change, engaging, opinion leaders, and champions. The development of psychometrically robust measures which can assess these constructs in public health and community settings may be a priority area of research for the field.

The most frequently addressed constructs appeared to fall within the ‘inner setting’ and ‘characteristics of individuals’ domains, suggesting that the focus of measures to date has been on understanding only the immediate environment where the innovation or intervention will be implemented. It appeared that measures addressing ‘outer setting’ or ‘process’ constructs were less frequently observed than other domains. The development of future measures should target these domains of the CFIR to ensure a greater breadth and depth of understanding of all factors which may influence the implementation of evidence into practice in public health and community settings.

### Comparison of the current review with the SIRC Instrument Review Project

Despite the similarity in review methodologies utilised by the current review and that undertaken by SIRC [16], few measures have been reported by both reviews. This is not surprising, as although the SIRC review captured some measures developed in education or workplace settings, other public health and community settings were not addressed. Furthermore, the SIRC review used a much broader inclusion criteria with regard to measures of CFIR constructs. For example, for the construct of ‘self-efficacy’, the SIRC review includes all measures of self-efficacy, regardless of the context in which self-efficacy is being examined. In contrast, the current review only includes measures which assess self-efficacy in the context of an individual’s perceived ability to implement the target innovation.

Despite these differences, the use of a common framework (CFIR) for examining constructs captured by different measures in the current review promotes consistency and complements the findings of the SIRC review.

### Limitations

It is possible that not all existing implementation measures in public health and community settings were captured by this review. The keywords used to identify measures were limited to ‘questionnaire’, ‘measure’, ‘scale’, or ‘tool’ and other possible terms such as ‘instrument’ and ‘test’ were not used. These terms were excluded due to the likelihood of identifying non-relevant publications related to clinical practice (e.g. surgical instruments, immunologic tests). However, the exclusion of these keywords may have meant that some relevant publications were not identified during the database search. Additionally, the review did not assess measures published in the grey literature and only studies published in English were included. However, it is likely that those measures which were identified represent the best available evidence, given their publication in peer-reviewed journals and indexing in four scientific databases. The psychometric properties that were chosen to be extracted from publications about each measure may have also limited the findings. For example, for studies that utilised CFA, only data pertaining to the RMSEA and CFI were recorded based on recommendations by Schmitt [32]. Included publications may have reported additional CFA metrics (such as goodness of fit (GFI) or the normed fit index (NI)); however, they were not included in this review.

Despite these limitations, the findings from this review are likely to be of value to public health researchers who are looking to identify measures with robust psychometric properties that can be used to assess implementation constructs. There are, however, a small number of constructs for which no measure could be identified. Developing measures which can assess these five remaining constructs will be an important consideration for future research.

## Conclusion

Existing measures of implementation constructs for use in public health and community settings require additional testing to enhance their reliability and validity. Further research is also needed to revalidate these measures in different settings and populations. At present, no single measure, or combination of measures, can be used to assess all constructs of the CFIR in public health and community settings. The development of new measures which can assess the broader range of implementation constructs across all of the CFIR domains should continue to be a priority for the field.

## Additional files

Additional file 1: Summary of the CFIR domains and constructs used to support mapping of included measure domains [19]. (DOCX 32.8 kb)
Additional file 2: Face and content validity of each measure [25, 40–45, 47–97, 120–221]. (DOCX 44.7 kb)
Additional file 3: Construct and criterion validity of each measure [25, 40–92, 94, 96, 222–228]. (DOCX 48.4 kb)
Additional file 4: Internal consistency and test-retest reliability of each measure [25, 40–45, 47–92, 94, 96]. (DOCX 45.7 kb)
Additional file 5: Responsiveness, acceptability, feasibility, and cross-cultural adaptation for each measure [38–45, 47–92, 94, 96, 98–112]. (DOCX 42.1 kb)
Additional file 6: Mapping of measure domains against the 37 CFIR constructs [38–45, 47–92, 94, 96]. (DOCX 60 kb)

## Abbreviations

CFA: confirmatory factor analysis
CFI: comparative fit index
CFIR: Consolidated Framework for Implementation Research
CINAHL: Cumulative Index to Nursing and Allied Health Literature
EFA: exploratory factor analysis
EMBASE: Excerpta Medica database
GFI: goodness of fit
IOF: Implementation Outcomes Framework
MEDLINE: Medical Literature Analysis and Retrieval System Online
MeSH: Medical Subject Headings
NF﻿I: normed fit index
PRISMA: Preferred Reporting Items for Systematic Reviews and Meta-Analyses
RMSEA: root mean square error of approximation
SIRC: Society for Implementation Research Collaboration

## References

[1] Davies P, Walker A, Grimshaw J (2010). A systematic review of the use of theory in the design of guideline dissemination and implementation strategies and interpretation of the results of rigorous evaluations. Implement Sci.

[2] Nilsen P (2015). Making sense of implementation theories, models and frameworks. Implement Sci.

[3] Tabak RG, Khoong EC, Chambers D, Brownson RC (2012). Bridging research and practice: models for dissemination and implementation research. Am J Prev Med.

[4] Martinez RG, Lewis CC, Weiner BJ (2014). Instrumentation issues in implementation science. Implement Sci.

[5] Rabin BA, Purcell P, Naveed S, Moser RP, Henton MD, Proctor EK, Brownson RC, Glasgow RE (2012). Advancing the application, quality and harmonization of implementation science measures. Implement Sci.

[6] Brennan SE, Bosch M, Buchan H, Green SE (2012). Measuring organizational and individual factors thought to influence the success of quality improvement in primary care: a systematic review of instruments. Implement Sci.

[7] Chaudoir SR, Dugan AG, Barr CH (2013). Measuring factors affecting implementation of health innovations: a systematic review of structural, organizational, provider, patient, and innovation level measures. Implement Sci.

[8] Chor KHB, Wisdom JP, Olin SCS, Hoagwood KE, Horwitz SM (2014). Measures for predictors of innovation adoption. Adm Policy Ment Health.

[9] Scott T, Mannion R, Davies H, Marshall M (2003). The quantitative measurement of organizational culture in health care: a review of the available instruments. Health Serv Res.

[10] Weiner BJ, Amick H, Lee SYD (2008). Conceptualization and measurement of organizational readiness for change: a review of the literature in health services research and other fields. Med Care Res Rev.

[11] King T, Byers JF (2007). A review of organizational culture instruments for nurse executives. J Nurs Adm.

[12] Emmons KM, Weiner B, Fernandez ME, Tu SP (2012). Systems antecedents for dissemination and implementation: a review and analysis of measures. Health Educ Behav.

[13] Squires JE, Estabrooks CA, Gustavsson P, Wallin L (2011). Individual determinants of research utilization by nurses: a systematic review update. Implement Sci.

[14] McDowell I (2006). Measuring health: a guide to rating scales and questionnaires.

[15] Hersen M (2006). Clinician’s handbook of adult behavioral assessment.

[16] Lewis CC, Fischer S, Weiner BJ, Stanick C, Kim M, Martinez RG (2015). Outcomes for implementation science: an enhanced systematic review of instruments using evidence-based rating criteria. Implement Sci.

[17] Lewis CC, Stanick CF, Martinez RG, Weiner BJ, Kim M, Barwick M, Comtois KA (2015). The Society for Implementation Research Collaboration Instrument Review Project: a methodology to promote rigorous evaluation. Implement Sci.

[18] Proctor E, Silmere H, Raghavan R, Hovmand P, Aarons G, Bunger A, Griffey R, Hensley M (2011). Outcomes for implementation research: conceptual distinctions, measurement challenges, and research agenda. Adm Policy Ment Health.

[19] Damschroder LJ, Aron DC, Keith RE, Kirsh SR, Alexander JA, Lowery JC (2009). Fostering implementation of health services research findings into practice: a consolidated framework for advancing implementation science. Implement Sci.

[20] The SIRC Instrument Review Project (IRP): A systematic review and synthesis of implementation science instruments [http://www.societyforimplementationresearchcollaboration.org/sirc-projects/sirc-instrument-project]

[21] Sampson M, McGowan J, Cogo E, Grimshaw J, Moher D, Lefebvre C (2009). An evidence-based practice guideline for the peer review of electronic search strategies. J Clin Epidemiol.

[22] Jenuwine E, Floyd J (2004). Comparison of Medical Subject Headings and text-word searches in MEDLINE to retrieve studies on sleep in healthy individuals. J Med Libr Assoc.

[23] Clinton-McHarg T, Carey M, Sanson-Fisher R, Shakeshaft A, Rainbird K (2010). Measuring the psychosocial health of adolescent and young adult (AYA) cancer survivors: a critical review. Health Qual Life Outcomes.

[24] Tzelepis F, Rose SK, Sanson-Fisher RW, Clinton-McHarg T, Carey ML, Paul CL (2014). Are we missing the Institute of Medicine’s mark? A systematic review of patient-reported outcome measures assessing quality of patient-centred cancer care. BMC Cancer.

[25] American Educational Research Association, American Psychological Association, National Council on Measurement in Education (2014). Standards for educational and psychological testing.

[26] Mokkink L, Terwee C, Patrick D, Alonso J, Stratford P, Knol D, Bouter L, de Vet H (2010). The COSMIN checklist for assessing the methodological quality of studies on measurement properties of health status measurement instruments: an international Delphi study. Qual Life Res.

[27] Anastasi A, Urbina S (1997). Psychological testing.

[28] Lohr KN, Aaronson NK, Alonso J, Audrey-Burnam M, Patrick DL, Perrin EB, Roberts JS (1996). Evaluating quality-of-life and health status instruments: development of scientific review criteria. Clin Ther.

[29] Kaiser HF (1960). Directional statistical decisions. Psychol Rev.

[30] Tabachnick BG, Fidell LS (2013). Using multivariate statistics.

[31] Hu L, Bentler PM (1999). Cutoff criteria for fit indexes in covariance structure analysis: conventional criteria versus new alternatives. Struct Equation Model.

[32] Schmitt TA (2011). Current methodological considerations in exploratory and confirmatory factor analysis. J Psychoeduc Assess.

[33] Cohen J (1988). Statistical power analysis for the behavioral sciences.

[34] Rubin A, Bellamy J (2012). Practitioner’s guide to using research for evidence-based practice.

[35] Marx RG, Menezes A, Horovitz L, Jones EC, Warren RF (2003). A comparison of two time intervals for test-retest reliability of health status instruments. J Clin Epidemiol.

[36] Streiner DL, Norman GR (2008). Health measurement scales: a practical guide to their development and use.

[37] Pedhazur EJ, Schmelkin LP (1991). Measurement, design, and analysis: an integrated approach.

[38] Aldridge JM, Laugksch RC, Fraser BJ (2006). School-level environment and outcomes-based education in South Africa. Learn Environ Res.

[39] Bowen GL, Rose RA, Ware WB (2006). The reliability and validity of the school success profile learning organization measure. Eval Program Plann.

[40] Canfield JP, Teasley ML, Abell N, Randolph KA (2012). Validation of a Mckinney-Vento Act implementation scale. Res Soc Work Pract.

[41] Chatterji M (2001). Measuring leader perceptions of school readiness for reforms: use of an iterative model combining classical and Rasch methods. J Appl Meas.

[42] Deschesnes M, Trudeau F, Kebe M (2009). Psychometric properties of a scale focusing on perceived attributes of a health promoting school approach. Can J Public Health.

[43] Gingiss PL, Gottlieb NH, Brink SG (1994). Measuring cognitive characteristics associated with adoption and implementation of health innovations in schools. Am J Health Promot.

[44] Gingiss PL, Gottlieb NH, Brink SG (1994). Increasing teacher receptivity toward use of tobacco prevention education programs. J Drug Educ.

[45] Hayes DM (1992). Toward the development and validation of a curriculum coordinator Role-efficacy Belief Instrument for sexuality education. J Sex Educ Ther.

[46] Hume A, McIntosh K (2013). Construct validation of a measure to assess sustainability of school-wide behavior interventions. Psychol Sch.

[47] Kingery PM, Holcomb JD, Jibaja-Rusth M, Pruitt BE, Buckner WP (1994). The health teaching self-efficacy scale. J Health Educ.

[48] Lambert LG, Monroe A, Wolff L (2010). Mississippi elementary school teachers’ perspectives on providing nutrition competencies under the framework of their school wellness policy. J Nutr Educ Behav.

[49] McIntosh K, MacKay LD, Hume AE, Doolittle J, Vincent CG, Horner RH, Ervin RA (2011). Development and initial validation of a measure to assess factors related to sustainability of school-wide positive behavior support. J Posit Behav Interv.

[50] Mellin EA, Bronstein L, Anderson-Butcher D, Amorose AJ, Ball A, Green J (2010). Measuring interprofessional team collaboration in expanded school mental health: model refinement and scale development. J Interprof Care.

[51] Steckler A, Goodman RM, McLeroy KR, Davis S, Koch G (1992). Measuring the diffusion of innovative health promotion programs. Am J Health Promot.

[52] Tuytens M, Devos G (2009). Teachers’ perception of the new teacher evaluation policy: a validity study of the policy characteristics scale. Teach Teach Educ.

[53] Atkinson NL (2007). Developing a questionnaire to measure perceived attributes of eHealth innovations. Am J Health Behav.

[54] Chung KC (2014). Gender, culture and determinants of behavioural intents to adopt mobile commerce among the Y generation in transition economies: evidence from Kazakhstan. Behav Inf Technol.

[55] Chung K-C, Holdsworth DK (2012). Culture and behavioural intent to adopt mobile commerce among the Y Generation: comparative analyses between Kazakhstan, Morocco and Singapore. Young Consumers.

[56] Davis FD (1989). Perceived usefulness, perceived ease of use, and user acceptance of information technology. MIS Q.

[57] Pillay H, Irving K, McCrindle A (2006). Developing a diagnostic tool for assessing tertiary students’ readiness for online learning. Int J Learn Tech.

[58] Pillay H, Irving K, Tones M (2007). Validation of the diagnostic tool for assessing tertiary students’ readiness for online learning. High Educ Res Dev.

[59] Saeed KA, Abdinnour S (2013). Understanding post-adoption IS usage stages: an empirical assessment of self-service information systems. Inf Syst J.

[60] Talukder M, Quazi A (2011). The impact of social influence on individuals’ adoption of innovation. J Org Comp Elect Com.

[61] Fang Y, Yang S, Feng B, Ni Y, Zhang K (2011). Pharmacists’ perception of pharmaceutical care in community pharmacy: a questionnaire survey in Northwest China. Health Soc Care Community.

[62] Kansanaho HM, Puumalainen II, Varunki MM, Airaksinen MSA, Aslani P (2004). Attitudes of Finnish community pharmacists toward concordance. Ann Pharmacother.

[63] Roberts AS, Benrimoj SI, Chen TF, Williams KA, Aslani P (2008). Practice change in community pharmacy: quantification of facilitators. Ann Pharmacother.

[64] Cochran JK, Bromley ML, Swando MJ (2002). Sheriff’s deputies’ receptivity to organizational change. Policing.

[65] Taxman FS, Henderson C, Young D, Farrell J (2014). The impact of training interventions on organizational readiness to support innovations in juvenile justice offices. Adm Policy Ment Health.

[66] Christensson L, Unosson M, Bachrach-Lindstrom M, Ek AC (2003). Attitudes of nursing staff towards nutritional nursing care. Scand J Caring Sci.

[67] Randall R, Nielsen K, Tvedt SD (2009). The development of five scales to measure employees’ appraisals of organizational-level stress management interventions. Work Stress.

[68] Boothroyd RA, Greenbaum PE, Wang W, Kutash K, Friedman RM (2011). Development of a measure to assess the implementation of children’s systems of care: the systems of care implementation survey (SOCIS). J Behav Health Serv Res.

[69] Chang SE, Pan YHV (2011). Exploring factors influencing mobile users’ intention to adopt multimedia messaging service. Behav Inf Technol.

[70] Collis B, Pals N. A model for predicting an individual’s use of a telematics application for a learning-related purpose. Int J Educ Telecommunications. 2000;6:63-103.

[71] Collis B, Peters O, Pals N (2001). A model for predicting the educational use of information and communication technologies. Instruct Sci.

[72] Greenbaum PE, Wang W, Boothroyd R, Kutash K, Friedman RM (2011). Multilevel confirmatory factor analysis of the systems of care implementation survey (SOCIS). J Behav Health Serv Res.

[73] Hall GE, George AA, Rutherford WL (1977). Measuring stages of concern about innovation: a manual for use of the SOC questionnaire.

[74] Hall GE, George A, Rutherford W (1979). Measuring stages of concern about the innovation: a manual for use of the SoC questionnaire.

[75] Monthuy-Blanc J, Bouchard S, Maiano C, Seguin M (2013). Factors influencing mental health providers’ intention to use telepsychotherapy in first nations communities. Transcult Psychiatry.

[76] Bess KD, Perkins DD, McCown DL (2010). Testing a measure of organizational learning capacity and readiness for transformational change in human services. J Prev Interv Community.

[77] Bouckenooghe D, Devos G, Van den Broeck H (2009). Organizational change questionnaire-climate of change, processes, and readiness: development of a new instrument. J Psychol.

[78] Caldwell DF, O’Reilly CA (2003). The determinants of team-based innovation in organizations the role of social influence. Small Group Res.

[79] Chwelos P, Benbasat I, Dexter AS (2001). Research report: empirical test of an EDI adoption model. Inf Syst Res.

[80] Dahlan N, Ramayah T, Mei LL, Karagiannis D, Reimer U (2002). Readiness to adopt data mining technologies: an exploratory study of telecommunication employees in Malaysia. Practical aspects of knowledge management.

[81] Hanusaik N, O’Loughlin JL, Kishchuk N, Eyles J, Robinson K, Cameron R (2007). Building the backbone for organisational research in public health systems: development of measures of organisational capacity for chronic disease prevention. J Epidemiol Community Health.

[82] Holt DT, Armenakis AA, Feild HS, Harris SG (2007). Readiness for organizational change: the systematic development of a scale. J Appl Behav Sci.

[83] Judge TA, Thoresen CJ, Pucik V, Welbourne TM (1999). Managerial coping with organizational change: a dispositional perspective. J Appl Psychol.

[84] Jung J, Nitzsche A, Neumann M, Wirtz M, Kowalski C, Wasem J, Stieler-Lorenz B, Pfaff H (2010). The Worksite Health Promotion Capacity Instrument (WHPCI): development, validation and approaches for determining companies’ levels of health promotion capacity. BMC Public Health.

[85] Molla A, Licker PS (2005). eCommerce adoption in developing countries: a model and instrument. Inf Manage.

[86] Molla A, Licker PS (2005). Perceived e-readiness factors in e-commerce adoption: an empirical investigation in a developing country. Int J Electron Commerce.

[87] Moore GC, Benbasat I (1991). Development of an instrument to measure the perceptions of adopting an information technology innovation. Inf Syst Res.

[88] Peltier JW, Schibrowsky JA, Zhao Y (2009). Understanding the antecedents to the adoption of CRM technology by small entrepreneurs vs owner-managers. Int Small Bus J.

[89] Ravichandran T (2000). Swiftness and intensity of administrative innovation adoption: an empirical study of TQM in information systems. Decision Sci.

[90] Saffu K, Walker JH, Hinson R (2007). An empirical study of perceived strategic value and adoption constructs: the Ghanaian case. Manag Decis.

[91] Strating MMH, Nieboer AP (2010). Norms for creativity and implementation in healthcare teams: testing the group innovation inventory. Int J Qual Health Care.

[92] Zeitz G, Johannesson R, Ritchie JE (1997). An employee survey measuring total quality management practices and culture development and validation. Group Org Manag.

[93] Taxman FS, Young DW, Wiersema B, Rhodes A, Mitchell S (2007). The national criminal justice treatment practices survey: multilevel survey methods and procedures. J Subst Abuse Treat.

[94] Lin SP, Yang HY (2009). Exploring key factors in the choice of e-health using an asthma care mobile service model. Telemed e-Health.

[95] Davis FD, Bagozzi RP, Warshaw PR (1989). User acceptance of computer-technology—a comparison of 2 theoretical-models. Manage Sci.

[96] Venkatesh V, Davis FD (2000). A theoretical extension of the technology acceptance model: four longitudinal field studies. Manage Sci.

[97] Molla A, Licker PL, Khosrow-Pour M (2002). PERM: a model of eCommerce adoption in developing countries. Issues and trends of information technology management in contemporary organizations.

[98] Al-Hudhaif SA, Alkubeyyer A (2011). E-commerce adoption factors in Saudi Arabia. Int J Bus Manage.

[99] Aldridge JM, Fraser BJ (2016). Teachers’ views of their school climate and its relationship with teacher self-efficacy and job satisfaction. Learn Environ Res.

[100] Alharbi S, Drew S. Using the technology acceptance model in understanding academics’ behavioural intention to use learning management systems. Int J Adv Comput Sci Appl (IJACSA). 2014;5.

[101] Bailey DB, Palsha SA (1992). Qualities of the Stages of Concern Questionnaire and implications for educational innovations. J Educ Res.

[102] Berkowitz R, Bowen G, Benbenishty R, Powers JD (2013). A cross-cultural validity study of the school success profile learning organization measure in Israel. Child Schools.

[103] Canfield JP (2014). The McKinney-Vento Act implementation scale: a second validation study. J Child Poverty.

[104] Cheung D, Hattie J, Ng D (2001). Reexamining the Stages of Concern Questionnaire: a test of alternative models. J Educ Res.

[105] Christensson L, Bachrach-Lindstrom M (2009). Adapting “the staff attitudes to nutritional nursing care scale” to geriatric nursing care. J Nutr Health Aging.

[106] Cetinkaya B (2012). Understanding teachers in the midst of reform: teachers’ concerns about reformed sixth grade mathematics curriculum in Turkey. Eurasia J Math Sci Technol Educ.

[107] Godoe P, Johansen T (2012). Understanding adoption of new technologies: technology readiness and technology acceptance as an integrated concept. J Eur Psychol Stud.

[108] Knapp P, Raynor DK, Thistlethwaite JE, Jones MB (2009). A questionnaire to measure health practitioners’ attitudes to partnership in medicine taking: LATCon II. Health Expect.

[109] Richardson JW (2011). Technology adoption in Cambodia: measuring factors impacting adoption rates. J Int Dev.

[110] Shotsberger PG, Crawford AR (1999). On the elusive nature of measuring teacher change: an examination of the stages of concern questionnaire. Eval Res Educ.

[111] Tan J, Tyler K, Manica A (2007). Business-to-business adoption of eCommerce in China. Inf Manage.

[112] Van Den Berg R, Ros A (1999). The permanent importance of the subjective reality of teachers during educational innovation: a concerns-based approach. Am Educ Res J.

[113] Edwards P, Roberts I, Clarke M, DiGuiseppi C, Pratap S, Wentz R, Kwan I, Cooper R (2007). Methods to increase response rates to postal questionnaires. Cochrane Database Syst Rev.

[114] Schafer J (1999). Multiple imputation: a primer. Stat Methods Med Res.

[115] Macfarlane A, O’Reilly-de Brun M, de Brun T, Dowrick C, O’Donnell C, Mair F, Spiegel W, van den Muijsenbergh M, van Weel Baumgarten E, Lionis C (2014). Healthcare for migrants, participatory health research and implementation science—better health policy and practice through inclusion. The RESTORE project. Eur J Gen Pract.

[116] Husted JA, Cook RJ, Farewell VT, Gladman DD (2000). Methods for assessing responsiveness: a critical review and recommendations. J Clin Epidemiol.

[117] Guyatt G, Walter S, Norman G (1987). Measuring change over time—assessing the usefulness of evaluative instruments. J Chronic Dis.

[118] Groves RM (2006). Nonresponse rates and nonresponse bias in household surveys. Public Opin Q.

[119] Armstrong JS, Overton TS (1977). Estimating nonresponse bias in mail surveys. J Mark Res.

[120] Rogers EM (1983). Diffusion of innovations.

[121] Hall GE, Hord SM (1987). Change in schools: facilitating the process.

[122] Bandura A (1986). Social foundations of thought and action: a social cognitive theory.

[123] Bronstein LR (2003). A model for interdisciplinary collaboration. Soc Work.

[124] Bronstein LR (2002). Index of interdisciplinary collaboration. Soc Work Res.

[125] Litwin G, Stringer R (1969). Motivation and organizational climate. Manage Int Rev.

[126] Taylor J, Bowers D (1972). Survey of organizations: a machine scored standardized questionnaire.

[127] Greenhalgh T, Robert G, Macfarlane F, Bate P, Kyriakidou O (2004). Diffusion of innovations in service organizations: systematic review and recommendations. Milbank Q.

[128] Oldenburg B, Parcel GS, Glanz K, Rimer BK, Viswanath K (2002). Diffusion of innovations. Health behavior and health education: theory, research, and practice.

[129] Goldman KD (1994). Perceptions of innovations as predictors of implementation levels: the diffusion of a nation wide health education campaign. Health Educ Behav.

[130] Parcel GS, O’Hara-Tompkins NM, Harrist RB, Basen-Engquist KM, McCormick LK, Gottlieb NH, Eriksen MP (1995). Diffusion of an effective tobacco prevention program. Part II: evaluation of the adoption phase. Health Educ Res.

[131] Lafferty CK. Diffusion of an asset building innovation in three Portage County school districts: A model of individual change. Kent: Kent State University; 2001.

[132] Fullan M (2001). The new meaning of educational change.

[133] Bandura A, McClelland DC (1977). Social learning theory.

[134] Gibson S, Dembo MH (1984). Teacher efficacy—a construct-validation. J Educ Psychol.

[135] Lewin K (1951). Field theory in social science: selected theoretical papers.

[136] Moos RH (1974). The social climate scales: an overview.

[137] Fisher DL, Fraser BJ (1991). School climate and teacher professional development. South Pac J Teach Educ.

[138] Fisher DL, Fraser BJ (1991). Validity and use of school environment instruments. J Classroom Interact.

[139] Bowen GL (1997). Organizational culture profile.

[140] Orthner DK, Cook PC, Sabah Y, Rosenfeld J (2005). Measuring organizational learning in human services. Development and validation of the organizational learning capacity assessment.

[141] Cameron KS, Bright D, Caza A (2004). Exploring the relationships between organizational virtuousness and performance. Am Behav Sci.

[142] McIntosh K, Horner RH, Sugai G, Sailor W, Dunlap G, Sugai G, Horner RH (2009). Sustainability of systems-level evidence-based practices in schools: current knowledge and future directions. Handbook of positive behavior support.

[143] Nysveen H, Pedersen PE, Thorbjornsen H (2005). Intentions to use mobile services: antecedents and cross-service comparisons. J Acad Mark Sci.

[144] Hupcey JE, Penrod J, Morse JM, Mitcham C (2001). An exploration and advancement of the concept of trust. J Adv Nurs.

[145] Bauer HH, Barnes SJ, Reichardt T, Neumann MM (2005). Driving consumer acceptance of mobile marketing: a theoretical framework and empirical study. J Electron Commerce Res.

[146] Hofstede G (1980). Culture’s consequences: international differences in work-related values.

[147] Yoo B, Donthu N (2002). The effects of marketing education and individual cultural values on marketing ethics of students. J Mark Educ.

[148] Bolton T (1983). Perceptual factors that influence the adoption of videotex technology: results of the channel 2000 field test. J Broadcasting.

[149] Bandura A (1982). Self-efficacy mechanism in human agency. Am Psychol.

[150] Beach LR, Mitchell TR (1978). A contingency model for the selection of decision strategies. Acad Manage Rev.

[151] Johnson EJ, Payne JW (1985). Effort and accuracy in choice. Manage Sci.

[152] Payne JW (1982). Contingent decision behavior. Psychol Bull.

[153] Swanson EB (1982). Measuring user attitudes in MIS research: a review. Omega.

[154] Swanson EB (1987). Information channel disposition and use. Decision Sci.

[155] Saga VL, Zmud RW, Levine L (1993). The nature and determinants of IT acceptance, routinization, and infusion. Diffusion, transfer, and implementation of information technology.

[156] Fishbein M (1979). A theory of reasoned action: some applications and implications. Nebr Symp Motiv.

[157] Frambach RT, Schillewaert N (2002). Organizational innovation adoption—a multi-level framework of determinants and opportunities for future research. J Bus Res.

[158] Venkatesh V, Morris MG, Davis GB, Davis FD (2003). User acceptance of information technology: toward a unified view. MIS Q.

[159] Igbaria M, Guimaraes T, Davis G (1995). Testing the antecedents of microcomputer usage via a structural equation model. J Manage Inf Syst.

[160] Igbaria M, Zinatelli N, Cragg P, Cavaye A (1997). Personal computing acceptance factors in small firms: a structural equation model. MIS Q.

[161] Al-Gahtani SS, King M (1999). Attitudes, satisfaction and usage: factors contributing to each in the acceptance of information technology. Behav Inf Technol.

[162] Taylor S, Todd PA (1995). Understanding information technology usage—a test of competing models. Inf Syst Res.

[163] Lam T, Cho V, Qu H (2007). A study of hotel employee behavioral intentions towards adoption of information technology. Int J Hosp Manag.

[164] Lewis W, Agarwal R, Sambamurthy V (2003). Sources of influence on beliefs about information technology use: an empirical study of knowledge workers. MIS Q.

[165] Selwyn N (1997). Students’ attitudes toward computers: validation of a computer attitude scale for 16-19 education. Comput Educ.

[166] Huang HM (2002). Student perceptions in an online mediated environment. Int J Instr Media.

[167] Watkins R, Leigh D, Triner D (2004). Assessing readiness for e-learning. Perform Improv Q.

[168] Smith PJ, Murphy KL, Mahoney SE (2003). Towards identifying factors underlying readiness for online learning: an exploratory study. Distance Educ.

[169] Smith PJ (2005). Learning preferences and readiness for online learning. Educ Psychol.

[170] Muse HE (2003). The web-based community college student: an examination of factors that lead to success and risk. Internet Higher Educ.

[171] Osborn V (2001). Identifying at-risk students in videoconferencing and web-based distance education. Am J Distance Educ.

[172] Roblyer MD, Marshall JC (2002). Predicting success of virtual high school students: preliminary results from an educational success prediction instrument. J Res Comput Educ.

[173] Scott WR (1998). Organizations: rational, natural, and open systems.

[174] Benrimoj SI, Roberts AS (2005). Providing patient care in community pharmacies in Australia. Ann Pharmacother.

[175] Roberts AS, Benrimoj SIC, Chen TF, Williams KA, Hopp TR, Aslani P (2005). Understanding practice change in community pharmacy: a qualitative study in Australia. Res Soc Adm Pharm.

[176] Raynor D, Thistlethwaite J, Hart K, Knapp P (2001). Are health professionals ready for the new philosophy of concordance in medicine taking?. Int J Pharm Pract.

[177] Hepler CD, Strand LM (1990). Opportunities and responsibilities in pharmaceutical care. Am J Hosp Pharm.

[178] Tesluk PE, Farr JL, Mathieu JE, Vance RJ (1995). Generalization of employee involvement training to the job setting: individual and situational effects. Pers Psychol.

[179] Orthner DK, Cook P, Sabah Y, Rosenfeld J (2006). Organizational learning: a cross-national pilot-test of effectiveness in children’s services. Eval Program Plann.

[180] Scott SG, Bruce RA (1994). Determinants of innovative behavior: a path model of individual innovation in the workplace. Acad Manage J.

[181] Bass BM, Avolio BJ (1994). Improving organizational effectiveness through transformational leadership.

[182] Arnold JA, Arad S, Rhoades JA, Drasgow F (2000). The empowering leadership questionnaire: the construction and validation of a new scale for measuring leader behaviors. J Organ Behav.

[183] Podsakoff PM, MacKenzie SB, Moorman RH, Fetter R (1990). Transformational leader behaviors and their effects on followers’ trust in leader, satisfaction, and organizational citizenship behaviors. Leadersh Q.

[184] Martino S, Ball SA, Gallon SL, Hall D, Garcia M, Ceperich S, Farentinos C, Hamilton J, Hausotter W (2006). Motivational interviewing assessment: supervisory tools for enhancing proficiency.

[185] Farrell J, Young DW, Taxman FS (2011). Effects of organizational factors on use of juvenile supervision practices. Crim Justice Behav.

[186] Caldwell DF, Chatman JA, O’Reilly CA (1990). Building organizational commitment: a multifirm study. J Occup Psychol.

[187] Saksvik P, Nytro K, Tvedt SD, Houdmont J, Leka S (2007). Healthy organizational change. Occupational health psychology: European perspectives on research, education and practice.

[188] Saksvik P, Tvedt SD, Nytro K, Andersen GR, Andersen TK, Buvik MP, Torvatn H (2007). Developing criteria for healthy organizational change. Work Stress.

[189] Fishbein M, Ajzen I (1975). Belief, attitude, intention, and behavior: an introduction to theory and research.

[190] Hartwick J, Barki H (1994). Explaining the role of user participation in information system use. Manage Sci.

[191] Hurt HT, Joseph K, Cook CD (1977). Scales for the measurement of innovativeness. Hum Commun Res.

[192] Zaltman G (1996). Metaphorically speaking: new technique uses multidisciplinary ideas to improve qualitative research. Mark Res.

[193] Hall GE, Wallace RD, Dossett WA (1973). A developmental conceptualization of the adoption process within educational institutions.

[194] Newlove BW, Hall GE (1976). A manual for assessing open-ended statements of concern.

[195] Davis FD (1993). User acceptance of information technology: system characteristics, user perceptions and behavioral impacts. Int J Man Mach Stud.

[196] Venkatesh V (2000). Determinants of perceived ease of use: integrating control, intrinsic motivation, and emotion into the technology acceptance model. Inf Syst Res.

[197] Venkatesh V, Davis FD (2000). Extrinsic and intrinsic motivation to use computers in the work place. J Appl Psychol.

[198] Kwon TH, Zmud RW, Boland RJ, Hirschheim R (1987). Unifying the fragmented models of information systems implementation. Critical issues in information systems research.

[199] Davenport TH, Harris JG, De Long DW, Jacobson AL (2001). Data to knowledge to results: building an analytic capability. Calif Manage Rev.

[200] Plsek PE (1997). Collaborating across organizational boundaries to improve the quality of care. Am J Infect Control.

[201] Kilo CM (1998). A framework for collaborative improvement: lessons from the Institute for Healthcare Improvement’s Breakthrough Series. Qual Manage Healthc.

[202] Kivimaki M, Elovainio M (1999). A short version of the team climate inventory: development and psychometric properties. J Occup Organ Psychol.

[203] Strating MMH, Nieboer AP (2009). Psychometric test of the team climate inventory-short version investigated in Dutch quality improvement teams. BMC Health Serv Res.

[204] Anderson N, West MA (1994). Team climate inventory: manual and user’s guide.

[205] Iacovou CL, Benbasat I, Dexter AS (1995). Electronic data interchange and small organizations: adoption and impact of technology. MIS Q.

[206] Raymond L, Pare G (1992). Measurement of information technology sophistication in small manufacturing businesses. Inf Resourc Manage J.

[207] Ferguson S. The benefits and barriers to adoption of EDI. Vancouver: University of British Columbia; 1992.

[208] Marsick VJ, Watkins KE (2003). Demonstrating the value of an organization’s learning culture: the dimensions of the learning organization questionnaire. Adv Dev Hum Resourc.

[209] Goodman RM, Speers MA, McLeroy K, Fawcett S, Kegler M, Parker E, Smith SR, Sterling TD, Wallerstein N (1998). Identifying and defining the dimensions of community capacity to provide a basis for measurement. Health Educ Behav.

[210] Crisp BR, Swerissen H, Duckett SJ (2000). Four approaches to capacity building in health: consequences for measurement and accountability. Health Promot Int.

[211] Hawe P, King L, Noort M, Jordens C, Lloyd B (2000). Indicators to help with capacity building in health promotion.

[212] Dean JW, Bowen DE (1994). Management theory and total quality—improving research and practice through theory development. Acad Manage Rev.

[213] Miller D, Friesen PH (1982). Innovation in conservative and entrepreneurial firms—2 models of strategic momentum. Strateg Manage J.

[214] Saraph JV, Benson PG, Schroeder RG (1989). An instrument for measuring the critical factors of quality management. Decision Sci.

[215] Van de Ven AH, Poole MS (1995). Explaining development and change in organizations. Acad Manage Rev.

[216] Hunt VD (1992). Quality in America: how to implement a competitive quality program.

[217] Grandzol JR. Implementing total quality: Critical relationships. Philadelphia: Temple University, Department of Management Science and Operations Management; 1996.

[218] Hackman JR, Oldham GR (1980). Work redesign.

[219] Schwartz R, Smith C, Speers MA, Dusenbury LJ, Bright F, Hedlund S, Wheeler F, Schmid TL (1993). Capacity building and resource needs of state health agencies to implement community-based cardiovascular disease programs. J Public Health Policy.

[220] Hawe P, Noort M, King L, Jordens C (1997). Multiplying health gains: the critical role of capacity-building within health promotion programs. Health Policy.

[221] Riley BL, Taylor SM, Elliott SJ (2001). Determinants of implementing heart health promotion activities in Ontario public health units: a social ecological perspective. Health Educ Res.

[222] Chatterji M, Sentovich C, Ferron J, Rendina-Gobioff G (2002). Using an iterative model to conceptualize, pilot test, and validate scores from an instrument measuring teacher readiness for educational reforms. Educ Psychol Meas.

[223] Pearlin LI, Menaghan EG, Lieberman MA, Mullan JT (1981). The stress process. J Health Soc Behav.

[224] Trumbo DA (1961). Individual and group correlates of attitudes toward work-related changes. J Appl Psychol.

[225] Miller VD, Johnson JR, Grau J (1994). Antecedents to willingness to participate in a planned organizational change. J Appl Commun Res.

[226] Mayer RC, Davis JH (1999). The effect of the performance appraisal system on trust for management: a field quasi-experiment. J Appl Psychol.

[227] Watson D, Clark LA, Tellegen A (1988). Development and validation of brief measures of positive and negative affect: the PANAS scales. J Pers Soc Psychol.

[228] Hong SM, Faedda S (1996). Refinement of the Hong psychological reactance scale. Educ Psychol Meas.

