# Supplementary material for: Psychometric properties of implementation measures for public health and community settings and mapping of constructs against the Consolidated Framework for Implementation Research: a systematic review
Source: Implement Sci. 2016 Nov 8;11:148. doi: 10.1186/s13012-016-0512-5 (PMC5100177; doi:10.1186/s13012-016-0512-5)
Supplement: Additional file 1: — Summary of the CFIR domains and constructs used to support mapping of included measure domains [19]. (DOCX 32.8 kb) [file 13012_2016_512_MOESM1_ESM.docx]

**Additional File 1.** Summary of the CFIR domains and constructs used to support mapping of included measure domains.*

| **Domain** | **Construct** | **Description** |
| --- | --- | --- |
| **Intervention Characteristics** | ***Intervention Source*** | Perceive the intervention has been **developed externally** or **internally** |
|  | ***Evidence Strength & Quality*** | Believe in the **quality and validity of evidence** supporting the intervention  Believe that the intervention will have the **desired outcomes** |
|  | ***Relative Advantage*** | Perceive there is an **advantage** to implementing the intervention  Perceive the intervention is **better than an alternative** solution |
|  | ***Adaptability*** | Degree to which the intervention can be **tailored or refined**  Degree to which the intervention can be **adapted to meet local needs** |
|  | ***Trialability*** | Degree to which the intervention can be **tested on a small scale**  Degree to which the intervention can be **reversed or stopped if necessary** |
|  | ***Complexity*** | **Perceived difficulty** of implementation e.g. duration, radicalness, disruptiveness, intricacy  **Number of steps required** to implement |
|  | ***Design Quality & Packaging*** | **Perceived excellence** of the intervention  How the intervention is **bundled, presented, and assembled** |
|  | ***Cost*** | **Costs** associated with implementing the intervention  Degree of **investment** in the intervention |
| **Outer Setting** | ***Client Needs & Resources*** | Knowledge of **barriers and facilitators to meeting client needs** |
|  | ***Cosmopolitanism*** | Degree to which the organisation is **networked with other external organisations** |
|  | ***Peer Pressure*** | **Competitive/peer pressure** to implement an intervention  Degree to which **other organisations have already implemented** the intervention |
|  | ***External Policy & Incentives*** | **External policies** e.g. legislation, mandates, regulations, recommendations, guidelines  **External incentives** e.g. pay-for-performance, public benchmark reporting |
| **Inner Setting** | ***Structural Characteristics*** | The **social architecture** of the organisation  The **age, maturity and size** of the organisation |
|  | ***Networks & Communications*** | The quality of **social networks and communication** within the organisation |
|  | ***Culture*** | **Norms and values** of the organisation |
|  | ***Implementation Climate*** | Degree to which **all individuals are receptive** to the intervention  Degree to which using the intervention is **rewarded, supported and expected** |
|  | ***Tension for Change*** | Perceive that the **current situation is intolerable**  Perceive that **change is needed** |
|  | ***Compatibility*** | How well the intervention **fits with** **individual’s norms, values and needs**  How well the intervention **fits with existing workflows and systems** |
|  | ***Relative Priority*** | **Perceived importance** of the implementation within the organisation |
|  | ***Organisational Incentives & Rewards*** | **Tangible organisational incentives** e.g. awards, promotions, raises  **Intangible organisational incentives** e.g. increased status or respect |
|  | ***Goals and Feedback*** | Degree to which **goals are clearly communicated to staff**  Degree to which **feedback is provided to staff** |
|  | ***Learning Climate*** | Staff **input and assistance** is requested by leaders, staff feel **essential and valued**  Staff feel it is **safe to try new methods**, staff have sufficient **time and space for reflection** **and evaluation** |
|  | ***Readiness for Implementation*** | Indicators that the **organisation is committed** to implement the intervention |
|  | ***Leadership Engagement*** | Indicators that **leaders and managers are committed** to implement the intervention  **Accountability of leaders and managers** to the success of the implementation |
|  | ***Available Resources*** | Degree to which **resources have been dedicated to the implementation** e.g. physical space, time, money, training  Degree to which **resources have been dedicated to on-going operations** |
|  | ***Access to Knowledge & Information*** | Ease with which staff can **access information and knowledge** about the intervention  Ease with which staff can **incorporate new information and knowledge** into existing work tasks |
| **Characteristics of Individuals** | ***Knowledge/Beliefs about Intervention*** | Individual **attitudes** towards the intervention, individual **value or importance** placed on the intervention  Individual **awareness of facts and truths** about the intervention. |
|  | ***Self-efficacy*** | Individual belief in their **ability to execute steps and actions** of the intervention  Individual belief in their **ability to achieve implementation goals** |
|  | ***Individual Stage of Change*** | Individual progress towards **skilled and sustained use** of the intervention |
|  | ***Identification with Organisation*** | Individual **perceptions of the organisation**  Individual **commitment to the organisation** |
|  | ***Other Personal Attributes*** | **Individual traits important for change** e.g. tolerance, motivation, intelligence, competence, learning style |
| **Process** | ***Planning*** | Degree to which implementation **behaviours and tasks are developed in advance**  **Quality of the behaviours and tasks** that have been developed |
|  | ***Engaging*** | **Attracting and encouraging individuals** to use of the intervention via education, role modeling, training, marketing |
|  | ***Opinion Leaders*** | Individuals within the organisation who can **influence the attitudes and beliefs of their colleagues** |
|  | ***Formally Appointed Internal Leaders*** | Individuals within the organisation **responsible for coordinating or project managing the intervention** |
|  | ***Champions*** | Individuals dedicated to **supporting, marketing, and driving** implementation  Individuals dedicated to **overcoming indifference or resistance** to implementation |
|  | ***External Change Agents*** | Individuals **appointed from an external entity to facilitate implementation** of the intervention |
|  | ***Executing*** | Implementation is carried out or **accomplished according to plan** |
|  | ***Reflecting & Evaluating*** | Quantitative and qualitative feedback about the **progress of the implementation**, and the **quality of the implementation**  Debriefing about the **experience of the implementation** |

**Adapted from Damschroder [19]- CFIR Constructs with short definitions.*
