# Supplementary material for: Psychometric properties of implementation measures for public health and community settings and mapping of constructs against the Consolidated Framework for Implementation Research: a systematic review
Source: Implement Sci. 2016 Nov 8;11:148. doi: 10.1186/s13012-016-0512-5 (PMC5100177; doi:10.1186/s13012-016-0512-5)
Supplement: Additional file 2: — Face and content validity of each measure [25, 40–45, 47–97, 120–221]. (DOCX 44.7 kb) [file 13012_2016_512_MOESM2_ESM.docx]

**Additional File 2.** Face and content validity of each measure.

| **Measure** | **Theory or framework**  ***(Standard 1.11)**** | **Items adapted from previous measure**  ***(Standard 1.11)**** | **Face/content validation**  ***(Standard 1.11)**** |
| --- | --- | --- | --- |
| **SCHOOLS** | | | |
| **Adopter Characteristics Scale**  [43] | Diffusion of Innovations  [120] | Not reported | Not reported |
| **Awareness and Concern Instrument**  [51] | Concerns Based Adoption Model  [74, 121] | Not reported | Pilot tested in two North Carolina school districts  Modified following pilot test |
| **HTSE Scale**  Health Teaching Self-efficacy Scale  [47] | Social Cognitive Theory  [122] | Internal Health Teaching Locus of Control;  External Health Teaching Locus of Control; Strategies Used in the Health Classroom; Skills Taught in the Health Classroom; Knowledge Imparted in the Health Classroom | Literature review  Measures validated in previous research in a similar setting |
| **IITC-ESMH**  Index of Inter-professional Team Collaboration – Expanded School Mental Health  [50] | Model for Interdisciplinary Collaboration  [123] | Index of Interdisciplinary Collaboration  [124] | Literature review  Tested for content validity and pilot tested with geographically diverse experts including researchers, practitioners, policy makers, graduate students (n = 21)  Item deletion, item addition, clarity improvement |
| **MVAIS**  McKinney-Vento Act Implementation Scale  [40] | Not reported | Not reported | Literature review  Policy provisions used to outline core constructs  Items reviewed by social work and education researchers for content validity and duplication |
| **Organisational Climate Instrument**  [51] | Not reported | Organisational Climate Questionnaire  [125]  Survey of Organisations  [126] | Pilot tested in two North Carolina school districts  Modified following pilot test |
| **Perceived Attributes of the Healthy Schools Approach Scale**  [42] | Diffusion of Innovations  [120, 127, 128] | Diffusion of Innovations Measures  [129-131] | Pilot-tested with school principals and school health promotion delegates (n = 8)  Modified following pilot test |
| **Policy Characteristics Scale**  [52] | Theory on Policy Implementation  [132] | Not reported | Literature review  Revised by school leaders and teachers |
| **REBI**  Role-Efficacy Belief Instrument  [45] | Social Learning Theory  [133] | Teacher Efficacy Scale  [134] | Literature review  Assessed by content area experts  Items judged as inappropriate removed |
| **Rogers’s Adoption Questionnaire**  [51] | Diffusion of Innovations  [120] | Not reported | Pilot tested in two North Carolina school districts  Modified following pilot test |
| **School WPI**  School Wellness Policy Instrument  [48] | Organisational Change Theory  [135] | Not reported | Literature review  Input from 3 researchers with expertise and experience in nutrition and elementary education  Pilot tested elementary school teachers (n = 43) |
| **SLEQ-SA**  School-level Environment Questionnaire – South Africa  [38] | Social Climate Dimensions  [136] | School-level Environment Questionnaire  [137, 138] | Literature review  Interviews with school management teams and teachers  Pilot tested with teachers  Refinement of scales and items |
| **SSP-LO Measure**  School Success Profile – Learning Organisation Measure  [39] | Not reported | Measure of Organisational Culture  [139]  Measure of Learning Organisations  [140]  Measure of Organisational Virtuousness  [141] | Literature review  Pilot tested in two middle schools and one high school  Reviewed by school officials, principals, specialists and teachers for wording and content |
| **SRR-LQ**  School Readiness for Reforms – Leader Questionnaire  [41] | Not reported | Not reported | Literature review  Reviewed by expert panel including school administrators and measurement/research specialists (n = 9)  Item rewording and reduction  Pilot tested with school leaders and administrators (n = 19) |
| **SUBSIST**  School-wide Universal Behaviour Sustainability Index – School Teams  [49] | Sustainability Model  [142] | Not reported | Literature review  Expert panel reviewed measure and completed a series of analyses (n = 21)  Pilot tested with School-Wide Positive Behavior Support (SWPBS) school team leaders and coaches (n = 25) |
| **Teacher Receptivity Measure**  [44] | Not reported | Not reported | Pre-tested with teachers (n = 116) and school nurses (n = 126) attending local and state-level professional meetings |
| **UNIVERSITIES/COLLEGES** | | | |
| **Intention to Adopt Mobile Commerce Questionnaire**  [54, 55] | Diffusion of Innovations  [120] | Behavioural Intention items  [143]  Trustworthiness items  [144]  Perceived Risk items  [145]  Cultural items  [146, 147] | Literature review  Professionally translated and back translated into Russian  Bilingual academics discussed inconsistencies and revised items (n = 5)  Pilot tested with university students (n = 30) |
| **Perceived Attributes of eHealth Innovations Questionnaire**  [53] | Diffusion of Innovations  [120] | Perceived Attributes of a Videotex System  [148] | Items reworded |
| **Perceived Usefulness and Ease of Use Scale**  [56] | Self-efficacy Theory  [149]  Cost-benefit Paradigm  [150-152]  Channel Disposition Model  [153, 154] | Not reported | Literature review  Pre-test interviews with experienced computer users from the School of Management including secretaries, students and professional staff (n = 15)  Item reduction |
| **Post-adoption Information Systems Usage Measure**  [59] | IT Acceptance, Routinisation, and Infusion  [155]  Conceptualisation of Post-adoptive Behavior  [151]  Technology Acceptance Model  [96] | Not reported | Literature review  Focus group with student users of self-service information systems (SIS) (n = 6)  Certain variables measured using existing scales from the literature  Judges conducted q-sorting exercise on the items based on construct deﬁnitions provided  Large scale data collection conducted to validate constructs |
| **Social Influence on Innovation Adoption Scale**  [60] | Theory of Reasoned Action  [156]  Technology Acceptance Model  [56]  Organisational Innovation Adoption Framework  [157]  Unified Theory of Acceptance and Use of Technology  [158] | Individual Adoption of Innovation  [159-161]  Attitude Toward Innovation  [161-163]  Peer Influence  [164]  Social Network  [164] | Expert review of sentence structure and content  Pilot tested to identify items that were misinterpreted or skipped over |
| **TSROL**  Tertiary Students Readiness for Online Learning Scale  [57, 58] | Not reported | Computer Attitude Scale  [165]  Student Perception of Online Environment  [166]  Online Leaner Readiness Self-assessment Instrument  [167]  Readiness for Online Learning Questionnaire  [168, 169]  Questionnaires to Asses Student Risk of Non-completion  [170, 171]  Educational Success Prediction Instrument  [172] | Literature review  Questionnaire administered to students, lecturers, tutors and computer help desk staff to determine key constructs |
| **PHARMACIES** | | | |
| **Facilitators of Practice Change Scale**  [63] | Organisational Theory  [173] | Third Agreement Programs  [174] | Literature review  Data from qualitative study [175]  Pilot mail survey of a random sample of Australian community pharmacies (n = 100) |
| **LATCon**  Leeds Attitude Towards Concordance Scale (Pharamacists)  [62] | Not reported | LATCon scale  [176] | Pilot tested in pharmacy students and practitioners  Evaluated by a scientist specialised in pharmacy ethics |
| **Perceived Barriers to the Provision of Pharmaceutical Care Questionnaire**  [61] | Pharmaceutical Care Model  [177] | Not reported | Discussed by a focus group  Pilot tested with community pharmacists (n = 10)  Feedback from two senior academic pharmacists |
| **POLICE/CORRECTIONAL FACILITIES** | | | |
| **Perceptions of Organisational Readiness for Change**  [65] | Not reported | Staff Cynicism for Change  [178]  National Criminal Justice Treatment Practices survey  [93, 179, 180]  Leadership of Immediate Supervisor  [181-183]  Comfort with Using Motivational Approaches  [184, 185]  Staff-Agency Value Concordance  [186] | Not reported |
| **Receptivity to Organisational Change Questionnaire**  [64] | Not reported | Not reported | Not reported |
| **NURSING HOMES** | | | |
| **IPM**  Intervention Process Measure  [67] | Healthy Change Process Index (HCPI)  [187, 188] | Change Process Healthiness Index  [188] | Literature review  Focus groups with employees |
| **SANN Scale**  Staff Attitudes to Nutritional Nursing Care Scale  [66] | Model for Attitude Studies  [189] | Not reported | Literature review  Tested by nurse aids (n = 65), working at one residential home in a municipality |
| **WHOLE COMMUNITIES/MULTIPLE SETTINGS** | | | |
| **4-E Telemeter**  [70, 71] | 4-E Model  [70] | Not reported | Literature review  Pilot tested |
| **Attitudes Towards Asthma Care Mobile Service Adoption Scale**  [94] | Technology Acceptance Model  [56] | Technology Acceptance Model measures  [56]  Subjective Norm  [190]  Innovativeness  [191] | Survey validation review process by experts from the field of healthcare industry  Measures adapted and reworded from validated sources |
| **Intention to Adopt Multimedia Messaging Service Scale**  [69] | Zaltman Metaphor Elicitation Technique [192] | Not reported | Literature review  One-on-one qualitative interviews |
| **SOCIS**  Systems of Care Implementation Survey  [68, 72] | Not reported | Not reported | Literature review  Consultation with expert panels and researchers in systems of care  Feedback from Board of Advisors, parents and professional leaders in children’s mental health |
| **SoCQ**  Stages of Concern Questionnaire  [73, 74] | Concerns-based Adoption Model  [193] | Open-ended Concerns Statement  [194] | Initial items edited for redundancy and reworded  Piloted with teachers and faculty staff (n = 359)  Interviews with teachers and faculty staff  Item reduction |
| **Telepsychotherapy Acceptance Questionnaire**  [75] | Technology Acceptance Model  [56] | User Acceptance of Information Technology  [195]  Determinants of Perceived Ease of Use  [196]  Motivation to Use Computers  [197] | Translated from French into English  Adapted from validated questionnaires |
| **OTHER WORKPLACES/ORGANISATIONS** | | | |
| **Adoption of Customer Relationship Management Technology Scale**  [88] | Diffusion of Innovations  [120]  Information Systems Adoption  [198]  Technology Acceptance Model  [56] | Not reported | Literature review  Interviews with co-op members who had purchased CRM systems and experts in the field  Feedback from an internal advisory committee  Items refined  Qualitative pilot study with store owners (n = 20) |
| **Coping with Organisational Change Scale**  [83] | Not reported | Not reported | Literature review Items administered to managers in the United States and Singapore  Item reduction |
| **DMRI**  Data Mining Readiness Index  [80] | Model for Building and Analytical Capability  [199] | Not reported | Literature review |
| **GII**  Group Innovation Inventory  [78, 91] | Institute of Healthcare Improvement ‘Breakthrough Series’ Approach  [200, 201] | Not reported | Literature review  Discussion groups with senior level managers  Translated into Dutch by two researchers independently [91]  Validated against the Team Climate Inventory (TCI) [202-204] |
| **Intention to Adopt Electronic Data Interchange Questionnaire**  [79] | Not reported | Perceived Benefits/Organisational Readiness/External Pressure/Competitive Pressure/Industry Pressure/ Financial Resources  [205]  IT Sophistication  [206]  Trading Partner Readiness  [207] | Literature review  Examined by colleague with expertise in measurement theory and questionnaire design  Pilot tested with members of the Purchasing Managers’ Association of Canada (PMAC) (n = 20)  Random probes used to test respondents’ understanding  Second pilot test with different group of PMAC members (n = 20) |
| **OCQ–C, P, R**  Organisational Change Questionnaire – Climate of Change, Processes, and Readiness  [77] | Not reported | Not reported | Literature review  Reviewed for clarity and redundancy  Reviewed by academic staff from a prominent business school (n = 10)  Pilot tested |
| **OLCS**  Organisational Learning Capacity Scale  [76] | Not reported | Dimensions of Learning Organisations Questionnaire  [208] | Items with redundant meanings were dropped |
| **Organisational Capacity Measure – Chronic Disease Prevention and Healthy Lifestyle Promotion**  [81] | Not reported | Items were adapted from earlier questionnaires designed to measure organisational practices/activities for (heart) health promotion  [209-211] | Literature review  Content review by expert researchers  Pre-testing through telephone interviews |
| **Organisational Environment and Processes Scale**  [89] | Total Quality Management  [212] | Environmental Dynamism Scale  [213]  Corporate Support for Quality and Management Support for Quality  [214] | Not reported |
| **PCI Scale**  Perceived Characteristics of Innovating Scale  [87] | Diffusion of Innovations  [120]  Technology Acceptance Model  [56] | Not reported | Literature review  Expert review (sorting procedure)  Pilot tested with business faculty at two universities (n = 20)  Second pilot test with utility company employees (n = 66)  Item reduction |
| **Perceived Strategic Value and Adoption of eCommerce Scale**  [90] | Technology Acceptance Model  [56] | Technology Acceptance Model  [56] | Literature review  Pilot tested with managers (n = 6) |
| **PERM Questionnaire**  Perceived eReadiness Model Questionnaire  [85, 86] | Perceived eReadiness Model  [97] | Not reported | Literature review  Interviews and informal discussions with academics and consultants with relevant experience (n = 6)  Reviewed and pre-tested by panel experts (n = 16)  Pilot tested in randomly selected business organisations in South Africa (n = 12) |
| **Readiness for Organisational Change Measure**  [82] | Teleological/Dialectical Change  [215] | Not reported | Literature review  Semi-structured interviews with senior and middle-level managers (n = 15)  Open-ended questionnaires completed by middle and lower-level managers (n = 60)  Review of list of themes by middle and lower-level managers (n = 291) |
| **TAM2 Scale**  Technology Acceptance Model 2 Scale  [96] | Technology Acceptance Model  [56] | Perceived Usefulness/Perceived Ease of Use/Behavioural Intention/Job Relevance/ Output Quality  [56, 95]  Subjective Norm  [162]  Result Demonstrability/ Image/Voluntariness  [87] | Focus groups with business professionals (n = 5) |
| **TQM and Culture Survey**  Total Quality Management and Culture Survey  [92] | Total Quality Management  [214, 216, 217] | Public Domain Scale  [216]  TQM surveys JDI scale  [218] | Literature review  Piloted with a class of university students |
| **WHPCI**  Worksite Health Promotion Capacity Instrument  [84] | Worksite Health Promotion Capacity Model  [219-221] | Not reported | Discussed by a focus group of organisational experts (n = 7)  Qualitative pre-tests with managing directors or decision-makers (n = 10) |

**Standard 1.11* – Describe the procedures followed in specifying and generating the test content [25].
