# Supplementary material for: Psychometric properties of implementation measures for public health and community settings and mapping of constructs against the Consolidated Framework for Implementation Research: a systematic review
Source: Implement Sci. 2016 Nov 8;11:148. doi: 10.1186/s13012-016-0512-5 (PMC5100177; doi:10.1186/s13012-016-0512-5)
Supplement: Additional file 3: — Construct and criterion validity of each measure [25, 40–92, 94, 96, 222–228]. (DOCX 48.4 kb) [file 13012_2016_512_MOESM3_ESM.docx]

**Additional File 3.** Construct and criterion validity of each measure.

|  | **Construct validity** | | | | | **Criterion validity** |
| --- | --- | --- | --- | --- | --- | --- |
| **Measure** | **Factor Analysis**  ***(Standard 1.13*)*** | **Items** | **Domains** | **Domain Descriptions** | **Convergent (*r*) >0.40**  **Divergent (*r*) < 0.30**  ***(Standard 1.16**)*** | **Known groups**  ***(Standard 1.20***)*** |
| **SCHOOLS** | | | | | | |
| **Adopter Characteristics Scale**  [43] | EFA  Eigenvalues > 1  3 factors represented 54% of variance | 18 | 3 | Innovativeness; Need for collegial support; Conservativeness | - | - |
| **Awareness and Concern Instrument**  [51] | EFA  Eigenvalues not reported  Variance not reported | 12 | 3 | Awareness; Concern; Interest | - | - |
| **HTSE Scale**  Health Teaching Self-efficacy Scale  [47] | EFA  Eigenvalues not reported  Variance not reported | 35 | 1 | Self-efficacy | - | **Able to discriminate between:**  Teachers with ≥ 5 years experience  Teachers with < 5 years experience  *(p=0.04)*  Teachers who taught health as a separate class  Teachers who taught health as an emphasis in another class  *(p=0.04)*  Teachers who spent at least 80% of time teaching health  Teachers who spent less than 80% of time teaching health  *(p=0.00)* |
| **IITC-ESMH**  Index of Inter-professional Team Collaboration – Expanded School Mental Health  [50] | EFA  Eigenvalues >1  4 factors represented 63% of variance | 26 | 4 | Reflection on process; Professional flexibility; Newly created professional activities; Role interdependence | - | - |
| **MVAIS**  McKinney-Vento Act Implementation Scale  [40] | CFA  RMSEA = 0.06  CFI = 0.97 | 26 | 3 | Preparation; Accessibility; Collaboration | - | - |
| **Organisational Climate Instrument**  [51] | EFA  Eigenvalues not reported  Variance not reported | 27 | 1 | Organisational climate | - | - |
| **Perceived Attributes of the Healthy Schools Approach Scale**  [42] | EFA  Eigenvalues >1  4 factors represented 65% of variance  CFA  RMSEA = 0.05  CFI = 0.95 | 14 | 4 | School contextual factors; Anticipated benefits; Collective efficacy; Relative advantages | - | - |
| **Policy Characteristics Scale**  [52] | EFA  Eigenvalues >1  3 factors represented 51% of variance  CFA  RMSEA = 0.08  CFI = 0.95 | 15 | 3 | Practicality; Need; Clarifying function | - | - |
| **REBI**  Role-Efficacy Belief Instrument  [45] | EFA  Eigenvalues not reported  Variance not reported | 12 | 2 | Personal role-efficacy belief; General role efficacy belief | - | - |
| **Rogers’s Adoption Questionnaire**  [51] | EFA  Eigenvalues not reported  Variance not reported | 9 | 3 | Relative advantage; Complexity; Observability | - | - |
| **School WPI**  School Wellness Policy Instrument  [48] | EFA  Eigenvalues >1  3 factors represented 64% of variance | 26 | 3 | Policy implementation (‘Moving Stage’); Policy support (‘Unfreezing Stage’); Policy acceptance (‘Refreezing Stage’) | - | - |
| **SLEQ-SA**  School-level Environment Questionnaire – South Africa  [38] | EFA  Eigenvalues not reported  7 factors represented 46% of variance | 51 | 7 | Parental involvement; Student support; Collegiality; Familiarity with OBE; Innovation; Resource adequacy; Work pressure | - | **Able to discriminate between:**  Outcome based education teachers  Non-outcome based education teachers  *(p<0.01)* |
| **SSP-LO Measure**  School Success Profile – Learning Organisation Measure  [39] | CFA  RMSEA = 0.05  CFI = 0.94 | 24 | 2 | Actions; Sentiments | - | - |
| **SRR-LQ**  School Readiness for Reforms – Leader Questionnaire  [41] | EFA  Eigenvalues >1  5 factors represented 92% of variance | 27 | 5 | Training needs; Impact; Resources; Implementation; Validity | **Convergent validity with:**  School Readiness for Reforms – Teacher Questionnaire  [222]  *(r = 0.17 to 0.22)* | - |
| **SUBSIST**  School-wide Universal Behaviour Sustainability Index – School Teams  [49] | EFA  Eigenvalues >1  4 factors represented 45% of variance  CFA  RMSEA = 0.04  CFI = 0.97 | 39 | 4 | School priority; District priority; Team use of data; Capacity building | - | - |
| **Teacher Receptivity Measure**  [44] | EFA  Eigenvalues >1  4 factors represented 41% of variance | 14 | 4 | General receptivity to tobacco prevention education; Support for teaching tobacco education; Personal involvement in teaching tobacco prevention education; School involvement with tobacco prevention | - | - |
| **UNIVERSITIES/COLLEGES** | | | | | | |
| **Intention to Adopt Mobile Commerce Questionnaire**  [54, 55] | EFA  Eigenvalues not reported  8 factors represented 73% of variance  CFA  RMSEA = 0.05  CFI = 0.93 | 35 | 8 | Perceived risk; Trustworthiness; Observability; Relative advantage; Compatibility; Complexity; Trialability; Behavioural intent | - | - |
| **Perceived Attributes of eHealth Innovations Questionnaire**  [53] | EFA  Eigenvalues >1  5 factors represented 55% of variance | 25 | 5 | Relative advantage; Simplicity; Trialability; Observability; Translatability | - | - |
| **Perceived Usefulness and Ease of Use Scale**  [56] | EFA  Eigenvalues not reported  Variance not reported | 12 | 2 | Perceived usefulness; Perceived ease of use | - | - |
| **Post-adoption Information Systems Usage Measure**  [59] | EFA  Eigenvalues not reported  Variance not reported  CFA  RMSEA = 0.05  CFI = 0.98 | 9 | 3 | Expanded usage; Integrative usage; Exploratory usage | - | **Able to discriminate between:** Users in routinisation stage  Users in infusion stage  Users in extension stage  *(p<0.05)* |
| **Social Influence on Innovation Adoption Scale**  [60] | EFA  Eigenvalues not reported  Variance not reported | 19 | 4 | Peer influence; Social network; Attitude towards innovation; Usage behaviour | - | - |
| **TSROL**  Tertiary Students Readiness for Online Learning Scale  [57, 58] | EFA  Eigenvalues >1  4 factors represented 63% of variance | 18 | 4 | Technical skills; Computer self-efficacy; Learner preferences; Attitudes towards computers | - | **Able to discriminate between:**  Aged >40 years  Aged up to 25 years  *(p <0.01)* |
| **PHARMACIES** | | | | | | |
| **Facilitators of Practice Change Scale**  [63] | EFA  Eigenvalues >1  7 factors represented 49% of variance | 35 | 7 | Relationship with physicians; Renumeration;  Pharmacy layout; Patent expectation; Manpower/staff; Communication/teamwork; External support/assistance | - | - |
| **LATCon**  Leeds Attitude Towards Concordance Scale (Pharamacists)  [62] | EFA  Eigenvalues not reported  3 factors represented 38% of variance | 12 | 3 | Respecting patient’s beliefs and coping strategies; Establishing therapeutic alliance; Sensitivity to patients’ reactions | - | - |
| **Perceived Barriers to the Provision of Pharmaceutical Care Questionnaire**  [61] | EFA  Eigenvalues >1  4 factors represented 65% of variance | 11 | 4 | Lack of external conditions for developing or providing pharmaceutical care; Lack of time and skills; Lack of information and economic incentive; Lack of support from other health professionals | - | - |
| **POLICE/CORRECTIONAL FACILITIES** | | | | | | |
| **Perceptions of Organisational Readiness for Change**  [65] | EFA  Eigenvalue >1  1 factor represented 66% of variance  CFA  RMSEA not reported  CFI not reported | - | 6 | Staff-Agency value concordance; Climate for learning; Formal and informal communication; Cynicism toward change; Supervisor leadership; Emphasis on case management activities | - | - |
| **Receptivity to Organisational Change Questionnaire**  [64] | EFA  Eigenvalues >1  Variance not reported | 149 | 7 | Receptivity to change; Crime control; Service; Traditionalism; Cynicism; Agency preparedness; Availability of resources | - | - |
| **NURSING HOMES** | | | | | | |
| **IPM**  Intervention Process Measure  [67] | EFA  Eigenvalues not reported  5 factors represented 67% of variance  CFA  RMSEA = 0.07  CFI = 0.93 | 24 | 5 | Line manager attitudes and actions; Exposure to intended intervention; Employee involvement; Employee readiness for change; Intervention history | - | - |
| **SANN Scale**  Staff Attitudes to Nutritional Nursing Care Scale  [66] | EFA  Eigenvalues > 1  5 factors represented 59% of variance | 19 | 5 | Self-ability; Individualisation; Importance of food; Assessment; Secured food intake | - | - |
| **WHOLE COMMUNITIES/MULTIPLE SETTINGS** | | | | | | |
| **4-E Telemeter**  [70, 71] | EFA  Eigenvalues >1  Variance not reported | 20 | 6 | Organisational aspects; Personal engagement - self-confidence; Effectiveness – learning; Effectiveness - long-term payoff; Ease-of-use - hardware/network; Ease-of-use - software | - | **Able to discriminate between:**  Instructors  Students  Research and support staff  *(p-value not reported)*  Elementary/secondary  University  Vocational/training  *(p-value not reported)*  Male  Female  *(p-value not reported)*  <30 years old  30-45 yearsold  >45 years old  *(p-value not reported)* |
| **Attitudes Towards Asthma Care Mobile Service Adoption Scale**  [94] | CFA  RMSEA = 0.06  CFI not reported | 27 | 6 | Perceived usefulness; Perceived ease of use; Attitude toward using; Behavioral intention; Subjective norm; Innovativeness | - | - |
| **Intention to Adopt Multimedia Messaging Service Scale**  [69] | EFA  Eigenvalues > 1  5 factors represented 64% of variance | 19 | 5 | Relative advantage; Facilitating conditions; Ease of use; Previous experience; Intention to use MMS | - | - |
| **SOCIS**  Systems of Care Implementation Survey  [68, 72] | CFA  RMSEA = 0.00 – 0.11  CFI = 0.90 – 1.00 | 70 | 14 | Family choice and voice; Individualised, comprehensive and culturally competent treatment; Outreach and access to care; Transformational leadership; Theory of change; Implementation plan; Local population of concern; Interagency and cross-sector collaboration; Values and principles; Comprehensive financial plan; Skilled provider network; Performance measurement system; Provider accountability; Management and governance | - | **Able to discriminate between:**  Grantees  Non-grantees  *(p<0.05)*  Mental health informants  Other informants (school, family, etc)  *(p<0.05)* |
| **SoCQ**  Stages of Concern Questionnaire  [73, 74] | EFA  Eigenvalues >1  7 factors represented more than 60% of variance | 35 | 7 | Awareness; Informational; Personal; Management; Consequence; Collaboration; Refocusing | - | - |
| **Telepsychotherapy Acceptance Questionnaire**  [75] | CFA  RMSEA = 0.09  CFI = 0.95 | 13 | 4 | Perceived usefulness; Perceived ease of use; Attitude toward videoconference; Intention to use | - | - |
| **OTHER WORKPLACES/ORGANISATIONS** | | | | | | |
| **Adoption of Customer Relationship Management Technology Scale**  [88] | EFA  Eigenvalues not reported  Variance not reported | 40 | 8 | Relative advantage; Product class knowledge; Environmental hostility; Environmental uncertainty or complexity; Switching costs; Personal risk orientation; Business change orientation; Interpersonal information seeking | - | - |
| **Coping with Organisational Change Scale**  [83] | CFA  RMSEA not reported  CFI = 0.94 | 12 | 1 | Coping with organisational change | - | - |
| **DMRI**  Data Mining Readiness Index  [80] | EFA  Eigenvalues ≥ 1  4 factors represented 66% of variance  EFA  Eigenvalues ≥ 1  2 factors represented 53% of variance | 28 | 6 | Clarity of the business strategy; Users’ skills and experience; Data-driven culture; Data quality Optimism; Innovativeness | - | - |
| **GII**  Group Innovation Inventory  [78, 91] | EFA  Eigenvalues > 1  Variance not reported  CFA  RMSEA = 0.05  CFI = 0.95 | 19 | 4 | Group functioning; Speed of action; Support for risk taking; Tolerance of mistakes | - | - |
| **Intention to Adopt Electronic Data Interchange Questionnaire**  [79] | EFA  Eigenvalues not reported  Variance not reported | 49 | 9 | Intent to adopt; Competitive pressure; Dependency on trading partner; Enacted trading partner power; Financial resources; Industry pressure; IT sophistication; Perceived benefits; Trading partner readiness | - | - |
| **OCQ–C, P, R**  Organisational Change Questionnaire – Climate of Change, Processes, and Readiness  [77] | EFA  Eigenvalues ≥ 1  3 factors represented 43% of variance  CFA  RMSEA < 0.08  CFI > 0.90 | 63 | 10 | Quality of change communication; Participation; Attitude of top management toward organizational change; Support of supervisors; Trust in leadership; Cohesion; Politicking; Emotional dimension of readiness for change; Cognitive dimension of readiness for change; Intentional dimension of readiness for change | - | **Able to discriminate between:**  For-profit organisation employees  Not-for-profit organisation employees  *(p-value not reported)*  Managers  Non-managers  *(p-value not reported)* |
| **OLCS**  Organisational Learning Capacity Scale  [76] | EFA  Eigenvalues not reported  Variance not reported | 16 | 6 | Practices to promote external alignment; Practices to promote internal alignment; Open communication practices; Learning practices; Practices of staff empowerment; Practice of supporting staff development | - | - |
| **Organisational Capacity Measure – Chronic Disease Prevention and Healthy Lifestyle Promotion**  [81] | EFA  Eigenvalues not reported  Variance not reported | 104 | 20 | *Organisation Support* -Managerial; Staff; Evaluation; Partnerships -  Effectiveness; *Skills to address* - Social determinants of health; Population needs assessment; Identify relevant practices; Planning; Implementation strategies; Evaluation; *Resources* -Adequacy; *Facilitators* - Internal; Resources; Government priority; Public priority; *Level of involvement* - SDH; Population needs assessment; Identify relevant practices; Planning; Evaluation | - | - |
| **Organisational Environment and Processes Scale**  [89] | EFA  Eigenvalues > 1  3 factors represented 65% of variance | 13 | 3 | Environmental dynamism; Quality orientation; IS management support | - | - |
| **PCI Scale**  Perceived Characteristics of Innovating Scale  [87] | EFA  Eigenvalues >1  7 factors represented 63% of variance | 25 | 8 | Relative advantage; Compatibility; Ease of use; Result demonstrability; Image; Visibility; Trialability; Voluntariness | - | **Able to discriminate between:**  Adopters  Non-adopters  *(p < 0.05)* |
| **Perceived Strategic Value and Adoption of eCommerce Scale**  [90] | *Perceived Strategic Value*  EFA  Eigenvalues > 1  4 factors represented 65% of variance  *Adoption of eCommerce*  EFA  Eigenvalues > 1  5 factors represented 79% of variance | 31 | 9 | Strategic decision support;  Information management;  Organisation support; Decision aid; Perceived usefulness; Perceived ease of use; Compatibility;  Organisational readiness;  External pressure | - | - |
| **PERM Questionnaire**  Perceived eReadiness Model Questionnaire  [85, 86] | EFA  Eigenvalues ≥ 1  Variance not reported | 70 | 11 | Awareness; Commitment; Human resources; Technological resources; Business resources; Governance; Government eReadiness; Market forces eReadiness; Supporting industries eReadiness; Initial eCommerce adoption; Institutionalisation of eCommerce | - | - |
| **Readiness for Organisational Change Measure**  [82] | EFA  Eigenvalues > 1  4 factors represented 61% of variance  CFA  RMSEA = 0.08  CFI = 0.98 | 25 | 4 | Appropriateness; Management support; Change efficacy; Personal valence | **Convergent validity with:**  Internal Mastery Scale  [223]  *(r = 0.26 to 0.46)*  General Attitudes Towards Change  [224]  *(r = 0.22 to 0.40)*  Perceptions of Organisation’s Communication Climate  [225]  *(r = 0.34 to 0.48)*  Perceived Ability of Management  [226]  *(r = 0.46 to 0.68)*  **Divergent validity with:** Negative Affect Schedule  [227]  *(r = -0.07 to -0.24)*  Rebelliousness  [228]  *(r = -0.19 to -0.37)* | - |
| **TAM2 Scale**  Technology Acceptance Model 2 Scale  [96] | EFA  Eigenvalues not reported  Variance not reported | 23 | 8 | Intention to use; Perceived usefulness; Perceived ease of use; Subjective norm; Image; Job relevance; Output quality; Result demonstrability | - | - |
| **TQM and Culture Survey**  Total Quality Management and Culture Survey  [92] | EFA  Eigenvalues > 1  Variance not reported  CFA  RMSEA not reported  CFI not reported | 56 | 12 | Management support; Suppliers; Data use; Suggestions; Improvement;  Customers; Supervisors; Job challenge; Cohesion; Communication; Innovation  Trust | - | - |
| **WHPCI**  Worksite Health Promotion Capacity Instrument  [84] | EFA  Eigenvalues >1  2 factors represented 77% of variance | 9 | 2 | Health promotion willingness; Health promotion management | - | - |

**Standard 1.13* – Describe relationships among test items or parts of the test by providing evidence concerning internal structure [25].

***Standard 1.16* – Provide evidence regarding relationships with conceptually related constructs (e.g. relationships with scores on other tests, and the degree to which these relationships are consistent with the underlying construct being measured) [25].

****Standard 1.20* – For effect-size measures used to draw inferences (e.g. mean test score differences between subgroups, correlation between test scores and criterion measures), report indices of the degree of uncertainty (e.g. significance tests, standard errors and confidence intervals) [25].
