# Supplementary material for: Psychometric properties of implementation measures for public health and community settings and mapping of constructs against the Consolidated Framework for Implementation Research: a systematic review
Source: Implement Sci. 2016 Nov 8;11:148. doi: 10.1186/s13012-016-0512-5 (PMC5100177; doi:10.1186/s13012-016-0512-5)
Supplement: Additional file 4: — Internal consistency and test-retest reliability of each measure [25, 40–45, 47–92, 94, 96]. (DOCX 45.7 kb) [file 13012_2016_512_MOESM4_ESM.docx]

**Additional File 4.** Internal consistency and test-retest reliability of each measure.

|  | | **Internal consistency** | | **Test-retest reliability** | | | | | |
| --- | --- | --- | --- | --- | --- | --- | --- | --- | --- |
| **Measure** | | **Cronbach’s alpha**  **(*α)* > 0.70**  **Kuder-Richardson**  **(KR-20) > 0.70**  ***(Standard 2.3*)*** | | **n** | | **Administration Period**  **(2-14 days)**  ***(Standard 2.19**)*** | | | **Cohen’s kappa**  **(*κ)* > 0.60**  **Pearson’s correlation**  **(*r)* > 0.70**  **Intra-Class Correlation**  **(ICC) > 0.70**  ***(Standard 2.19**)*** |
| **SCHOOLS** | | | | | | | | | |
| **Adopter Characteristics Scale**  [43] | | **Cronbach’s alpha**  **Total scale = Not reported**  Innovativeness = 0.83  Need for collegial support = 0.76  Conservativeness = 0.60 | | - | | - | | | - |
| **Awareness and Concern Instrument**  [51] | | **Cronbach’s alpha**  **Total scale = Not reported**  Awareness = 0.76  Concern = 0.72  Interest = 0.62 | | - | | - | | | - |
| **HTSE Scale**  Health Teaching Self-efficacy Scale  [47] | | **Cronbach’s alpha**  **Total scale = 0.96** | | 31 | | 14 days | | | Pearson’s correlation (r) = 0.82 |
| **IITC-ESMH**  Index of Inter-professional Team Collaboration – Expanded School Mental Health  [50] | | **Cronbach’s alpha**  **Total Scale = Not reported**  Reflection on process = 0.91  Professional flexibility = 0.91  Newly created professional activities = 0.84  Role interdependence = 0.80 | | - | | - | | | - |
| **MVAIS**  McKinney-Vento Act Implementation Scale  [40] | | **Cronbach’s alpha**  **Total scale = 0.94**  Preparation = 0.87  Accessibility = 0.91  Collaboration = 0.92 | | - | | - | | | **-** |
| **Organisational Climate Instrument**  [51] | | **Cronbach’s alpha**  **Total scale = 0.92** | | - | | - | | | - |
| **Perceived Attributes of the Healthy Schools Approach Scale**  [42] | | **Cronbach’s alpha**  **Total scale = Not reported**  School contextual factors = 0.85  Anticipated benefits = 0.77  Collective efficacy = 0.73  Relative advantages = 0.60 | | - | | - | | | - |
| **Policy Characteristics Scale**  [52] | | **Cronbach’s alpha**  **Total scale = Not reported**  Practicality = 0.86  Need = 0.80  Clarifying function = 0.71 | | - | | - | | | - |
| **REBI**  Role-Efficacy Belief Instrument  [45] | | **Cronbach’s alpha**  **Total scale = Not reported**  Personal role-efficacy belief = 0.81  General role-efficacy belief = 0.68 | | - | | - | | | - |
| **Rogers’s Adoption Questionnaire**  [51] | | **Cronbach’s alpha**  **Total scale = Not reported**  Relative advantage = 0.88  Complexity = 0.83  Observability = 0.77 | | - | | - | | | - |
| **School WPI**  School Wellness Policy Instrument  [48] | | **Cronbach’s alpha**  **Total scale = Not reported**  Moving Stage = 0.92  Unfreezing Stage = 0.86  Refreezing Stage = 0.86 | | - | | - | | | - |
| **SLEQ-SA**  School-level Environment Questionnaire – South Africa  [38] | | **Cronbach’s alpha**  **Total scale = Not reported**  Parental involvement = 0.86  Student support = 0.75  Collegiality = 0.92  Familiarity with OBE = 0.77  Innovation = 0.77  Resource adequacy = 0.77  Work pressure = 0.69 | | - | | - | | | - |
| **SSP-LO Measure**  School Success Profile – Learning Organisation Measure  [39] | | **Cronbach’s alpha**  **Total scale = Not reported**  Actions = 0.93  Sentiments = 0.95 | | - | | - | | | - |
| **SRR-LQ**  School Readiness for Reforms – Leader Questionnaire  [41] | | **Cronbach’s alpha**  **Total scale = Not reported**  Training needs = 0.89  Impact = 0.84  Resources = 0.79  Implementation = 0.76  Validity = 0.70 | | - | | - | | | - |
| **SUBSIST**  School-wide Universal Behaviour Sustainability Index – School Teams  [49] | | **Cronbach’s alpha**  **Total scale = 0.87** | | 21 | | 14 | | | Pearson’s correlation (r) = 0.96 |
| **Teacher Receptivity Measure**  [44] | | **Cronbach’s alpha**  **Total scale = Not reported**  General receptivity to tobacco prevention education = 0.08  Support for teaching tobacco education = 0.74  Personal involvement in teaching tobacco prevention education = 0.66  School involvement with tobacco prevention = 0.63 | | - | | - | | | - |
| **UNIVERSITIES/COLLEGES** | | | | | | | | | |
| **Intention to Adopt Mobile Commerce Questionnaire**  [54, 55] | | **Cronbach’s alpha**  **Total scale = Not reported**  Perceived risk = 0.86  Trustworthiness = 0.85  Observability = 0.73  Relative advantage = 0.79  Compatibility = 0.89  Complexity = 0.90  Trialability = 0.87  Behavioural intent = 0.79 | | - | | - | | | - |
| **Perceived Attributes of eHealth Innovations Questionnaire**  [53] | | **Cronbach’s alpha**  **Total scale = Not reported**  Relative advantage = 0.91  Simplicity = 0.85  Trialability = 0.66  Observability = 0.76  Translatability = 0.74 | | - | | - | | | - |
| **Perceived Usefulness and Ease of Use Scale**  [56] | | **Cronbach’s alpha**  **Total scale = Not reported**  Perceived usefulness = 0.98  Perceived ease of use = 0.94 | | - | | - | | | - |
| **Post-adoption Information Systems Usage Measure**  [59] | | **Cronbach’s alpha**  **Total scale = Not reported**  Expanded usage = 0.80  Integrative usage = 0.85  Exploratory usage = 0.80 | | - | | - | | | - |
| **Social Influence on Innovation Adoption Scale**  [60] | | **Cronbach’s alpha**  **Total scale = Not reported**  Peer influence = 0.76  Social network = 0.76  Attitude toward innovation = 0.92  Usage behaviour = 0.92 | | - | | - | | | - |
| **TSROL**  Tertiary Students Readiness for Online Learning Scale  [57, 58] | | **Cronbach’s alpha**  **Total scale = Not reported**  Technical skills = 0.92  Computer self-efficacy = 0.88  Learner preferences = 0.55  Attitudes towards computers = 0.76 | | - | | - | | | - |
| **PHARMACIES** | | | | | | | | | |
| **Facilitators of Practice Change Scale**  [63] | | **Cronbach’s alpha**  **Total scale = Not reported**  Relationship with physicians = 0.90  Renumeration = 0.82  Pharmacy layout = 0.81  Patent expectation = 0.82  Manpower/staff = 0.80  Communication/teamwork = 0.77  External support/assistance = 0.74 | | - | | - | | | - |
| **LATCon**  Leeds Attitude Towards Concordance Scale (Pharamacists)  [62] | | **Cronbach’s alpha**  **Total scale = Not reported**  Respecting patient’s beliefs and coping strategies = 0.60  Establishing therapeutic alliance = 0.65  Sensitivity to patients’ reactions = 0.66 | | - | | - | | | - |
| **Perceived Barriers to the Provision of Pharmaceutical Care Questionnaire**  [61] | | **Cronbach’s alpha**  **Total scale = 0.74**  Lack of external conditions for developing or providing pharmaceutical care = 0.71  Lack of time and skills = 0.72  Lack of information and economic incentive = 0.69  Lack of support from other health professionals = 0.74 | | - | | - | | | - |
| **POLICE/CORRECTIONAL FACILITIES** | | | | | | | | | |
| **Perceptions of Organisational Readiness for Change**  [65] | | **Cronbach’s alpha**  **Total scale = Not reported**  Staff-Agency value concordance = 0.91  Climate for learning = not reported  Formal and informal communication = not reported  Cynicism toward change = 0.90  Supervisor leadership = 0.66  Emphasis on case management activities = not reported | | - | | - | | | - |
| **Receptivity to Organisational Change Questionnaire**  [64] | | **Cronbach’s alpha**  **Total scale = Not reported**  Receptivity to change = 0.72  Crime control = 0.79  Service = 0.76  Traditionalism = 0.54  Cynicism = 0.83  Agency preparedness = 0.91  Availability of resources = 0.79 | | - | | - | | | - |
| **NURSING HOMES** | | | | | | | | | |
| **IPM**  Intervention Process Measure  [67] | | **Cronbach’s alpha**  **Total scale = Not reported**  Line manager attitudes and actions = 0.89  Exposure to intended intervention = 0.85  Employee involvement = 0.84  Employee readiness for change = 0.86  Intervention history = not reported | | - | | - | | | - |
| **SANN Scale**  Staff Attitudes to Nutritional Nursing Care Scale  [66] | | **Cronbach’s alpha**  **Total scale = 0.86**  Range for domains = 0.63-0.73 | | - | | - | | | - |
| **WHOLE COMMUNITIES/MULTIPLE SETTINGS** | | | | | | | | | |
| **4-E Telemeter**  [70, 71] | | **Cronbach’s alpha**  **Total Scale = Not reported** | | - | | - | | - | |
| **Attitudes Towards Asthma Care Mobile Service Adoption Scale**  [94] | | **Cronbach’s alpha**  **Total Scale = Not reported**  Range for domains = 0.80 - 0.95 | | - | | - | | - | |
| **Intention to Adopt Multimedia Messaging Service Scale**  [69] | | **Cronbach’s alpha**  **Total Scale = Not reported**  Relative advantage = 0.88  Facilitating conditions = 0.79  Ease of use = 0.85  Previous experience = 0.69  Intention to use MMS = 0.83 | | - | | - | | - | |
| **SOCIS**  Systems of Care Implementation Survey  [68, 72] | | **Cronbach’s alpha**  **Total scale = Not reported**  Range for domains = 0.69 - 0.94 | | - | | - | | - | |
| **SoCQ**  Stages of Concern Questionnaire  [73, 74] | | **Kuder-Richardson**  **Total scale = Not reported**  Awareness = 0.64  Informational = 0.78  Personal = 0.83  Management = 0.75  Consequence = 0.76  Collaboration = 0.82  Refocusing = 0.71 | | 132 | | 14 days | | **Pearson’s correlation**  Awareness (r) = 0.65  Informational (r) = 0.86  Personal (r) = 0.82  Management (r) = 0.81  Consequence (r) = 0.76  Collaboration (r) = 0.84  Refocusing (r) = 0.71 | |
| **Telepsychotherapy Acceptance Questionnaire**  [75] | | **Cronbach’s alpha**  **Total scale = 0.94** | | - | | - | | - | |
| **OTHER WORKPLACES/ORGANISATIONS** | | | | | | | | | |
| **Adoption of Customer Relationship Management Technology Scale**  [88] | **Cronbach’s alpha**  **Total scale = Not reported**  Relative advantage = 0.91  Product class knowledge = 0.91  Environmental hostility = 0.75  Environmental uncertainty/complexity = 0.75  Switching costs = 0.91  Personal risk orientation = 0.72  Business change orientation = 0.42  Interpersonal information seeking = 0.42 | | - | | - | | - | | |
| **Coping with Organisational Change Scale**  [83] | **Cronbach’s alpha**  **Total scale = 0.77** | | - | | - | | - | | |
| **DMRI**  Data Mining Readiness Index  [80] | **Cronbach’s alpha**  **Total scale = Not reported**  Clarity of the business strategy = 0.89  Users’ skills and experience = 0.86  Data-driven culture = 0.79  Data quality = 0.86  Optimism = 0.81  Innovativeness = 0.50 | | - | | - | | - | | |
| **GII**  Group Innovation Inventory  [78, 91] | **Cronbach’s alpha**  **Total scale = Not reported**  Group functioning = 0.79  Speed of action = 0.70  Support for risk taking = 0.64  Tolerance of mistakes = 0.45 | | - | | - | | - | | |
| **Intention to Adopt Electronic Data Interchange Questionnaire**  [79] | **Cronbach’s alpha (Fornell Statistic)**  **Total scale = Not reported**  Intent to adopt = 0.90  Competitive pressure = 0.76  Dependency on trading partner = 0.73  Enacted trading partner power = 0.83  Financial resources = 0.77  Industry pressure = 0.81  IT sophistication = 0.86  Perceived benefits = 0.94  Trading partner readiness = 0.82 | | - | | - | | - | | |
| **OCQ–C, P, R**  Organisational Change Questionnaire – Climate of Change, Processes, and Readiness  [77] | **Cronbach’s alpha**  **Total scale = Not reported**  Range for domains = 0.68 - 0.89 | | - | | - | | - | | |
| **OLCS**  Organisational Learning Capacity Scale  [76] | **Cronbach’s alpha**  **Total scale = 0.94**  Practices to promote external alignment = 0.78  Practices to promote internal alignment = 0.79  Open communication practices = 0.80  Learning practices = 0.80  Practices of staff empowerment = 0.74  Practices of supporting staff development = 0.84 | | - | | - | | - | | |
| **Organisational Capacity Measure – Chronic Disease Prevention and Healthy Lifestyle Promotion**  [81] | **Cronbach’s alpha**  **Total scale = Not reported**  Range for domains = 0.70 - 0.88 | | - | | - | | - | | |
| **Organisational Environment and Processes Scale**  [89] | **Cronbach’s alpha**  **Total scale = Not reported**  Environmental dynamism = 0.75  Quality orientation = 0.83  IS management support = 0.79 | | - | | - | | - | | |
| **PCI Scale**  Perceived Characteristics of Innovating Scale  [87] | **Cronbach’s alpha**  **Total scale = Not reported**  Relative advantage = 0.90  Compatibility = 0.86  Ease of use = 0.84  Result demonstrability = 0.79  Image = 0.79  Visibility = 0.83  Trialability = 0.71  Voluntariness = 0.82 | | - | | - | | - | | |
| **Perceived Strategic Value and Adoption of eCommerce Scale**  [90] | **Cronbach’s alpha**  **Total scale = Not reported**  Strategic decision support = 0.88  Information management = 0.80  Organisation support = 0.80  Decision aid = 0.70  Perceived usefulness = 0.96  Perceived ease of use = 0.93  Compatibility = 0.92  Organisational readiness = 0.81  External pressure = 0.81 | | - | | - | | - | | |
| **PERM Questionnaire**  Perceived eReadiness Model Questionnaire  [85, 86] | **Cronbach’s alpha**  **Total scale = Not reported**  Perceived organisational eReadiness = 0.93  Perceived external eReadiness = 0.79 | | - | | - | | - | | |
| **Readiness for Organisational Change Measure**  [82] | **Cronbach’s alpha**  **Total scale = Not reported**  Appropriateness = 0.94  Management support = 0.87  Change efficacy = 0.82  Personal valence = 0.66 | | - | | - | | - | | |
| **TAM2 Scale**  Technology Acceptance Model 2 Scale  [96] | **Cronbach’s alpha**  **Total scale = Not reported**  Intention to Use = 0.91  Perceived Usefulness = 0.93  Perceived Ease of Use = 0.93  Subjective Norm = 0.90  Image = 0.91  Job Relevance = 0.90  Output Quality = 0.88  Result Demonstrability = 0.89 | | - | | - | | - | | |
| **TQM and Culture Survey**  Total Quality Management and Culture Survey  [92] | **Cronbach’s alpha**  **Total scale = Not reported**  All domains > 0.65 | | - | | - | | - | | |
| **WHPCI**  Worksite Health Promotion Capacity Instrument  [84] | **Cronbach’s alpha**  **Total scale = Not reported**  Health promotion willingness = 0.83  Health promotion management = 0.91 | | - | | - | | - | | |

**Standard 2.3* – For each total score, subscore or combination of scores report estimates of relevant indices of reliability [25].

***Standard 2.19* – Describe each method of quantifying the reliability/precision of scores and use statistics appropriate to the method. (If the test-retest or alternate form approach is used, the interval between administrations should be indicated) [25].
