# Supplementary material for: Psychometric properties of implementation measures for public health and community settings and mapping of constructs against the Consolidated Framework for Implementation Research: a systematic review
Source: Implement Sci. 2016 Nov 8;11:148. doi: 10.1186/s13012-016-0512-5 (PMC5100177; doi:10.1186/s13012-016-0512-5)
Supplement: Additional file 5: — Responsiveness, acceptability, feasibility, and cross-cultural adaptation for each measure [38–45, 47–92, 94, 96, 98–112]. (DOCX 42.1 kb) [file 13012_2016_512_MOESM5_ESM.docx]

**Additional File 5.** Responsiveness, acceptability, feasibility and cross-cultural adaptation for each measure.

|  | Responsiveness | | Acceptability/feasibility |  |
| --- | --- | --- | --- | --- |
| Measure | Detects intervention  effect size > 0.5  *(Standard 13.3*)* | < 5% floor and ceiling effects  *(Standard 13.3*)* | Missing items  Time to complete  Time to interpret/score  *(Standard 4.2**)* | Revalidation/Cross-cultural Adaptation  *(Standard 7.6***)* |
| SCHOOLS | | | | |
| Adopter Characteristics Scale  [43] | - | - | - | - |
| Awareness and Concern Instrument  [51] | - | - | - | - |
| HTSE Scale  Health Teaching Self-efficacy Scale  [47] | Effect size ranged from 2.08 - 4.26 | - | - | - |
| IITC-ESMH  Index of Inter-professional Team Collaboration – Expanded School Mental Health  [50] | - | - | - | - |
| MVAIS  McKinney-Vento Act Implementation Scale  [40] | - | - | - | Culture/country – USA  Sample/setting – 228 school social workers  Construct validity – 4 factors, 26 items  [103] |
| Organisational Climate Instrument  [51] | - | - | - | - |
| Perceived Attributes of the Healthy Schools Approach Scale  [42] | - | - | - | - |
| Policy Characteristics Scale  [52] | - | - | Missing items < 1.5% | - |
| REBI  Role-Efficacy Belief Instrument  [45] | - | - | - | - |
| Rogers’s Adoption Questionnaire  [51] | - | - | - | - |
| School WPI  School Wellness Policy Instrument  [48] | - | - | - | - |
| SLEQ-SA  School-level Environment Questionnaire – South Africa  [38] | Effect size ranged from 2.05 - 4.99 | - | - | Culture/country – Australia  Sample/setting – 781 teachers from 29 high schools  Construct validity – 6 factors, 48 items  [99] |
| SSP-LO Measure  School Success Profile – Learning Organisation Measure  [39] | - | - | 15 minutes to complete | Culture/country – Israel  Sample/setting – 96 employees (homeroom teachers, teachers, assistants and professionals) from 3 junior high and high schools  Construct validity – 2 factors confirmed  [102] |
| SRR-LQ  School Readiness for Reforms – Leader Questionnaire  [41] | - | - | - | - |
| SUBSIST  School-wide Universal Behaviour Sustainability Index – School Teams  [49] | - | - | - | - |
| Teacher Receptivity Measure  [44] | - | - | - | - |
| UNIVERSITIES/COLLEGES | | | | |
| Intention to Adopt Mobile Commerce Questionnaire  [54, 55] | - | - | - | Culture/country – Morroco, Singapore, Kazakhstan  Sample/setting – 502 students from 6 tertiary education institutions  Construct validity – performed but not reported  [55] |
| Perceived Attributes of eHealth Innovations Questionnaire  [53] | - | - | - | - |
| Perceived Usefulness and Ease of Use Scale  [56] | - | - | - | Culture/country – USA  Sample/setting – 156 managers across four organisations  Construct validity – 8 factors, 23 items  [96] |
| Post-adoption Information Systems Usage Measure  [59] | - | - | Missing items = 3.4% | - |
| Social Influence on Innovation Adoption Scale  [60] | - | - | - | - |
| TSROL  Tertiary Students Readiness for Online Learning Scale  [57, 58] | - | - | - | - |
| PHARMACIES | | | | |
| Facilitators of Practice Change Scale  [63] | - | - | Missing items = 1.2% | - |
| LATCon  Leeds Attitude Towards Concordance Scale (Pharmacists)  [62] | - | - | - | Country/culture – UK  Sample/setting – UK medical students, nursing students, and pre-registration pharmacists.  Construct validity – 20 items, five factors  [108] |
| Perceived Barriers to the Provision of Pharmaceutical Care Questionnaire  [61] | - | - | - | - |
| POLICE/CORRECTIONAL FACILITIES | | | | |
| Perceptions of Organisational Readiness for Change  [65] | Effect size ranged from -0.60 - 0.69 | - | - | - |
| Receptivity to Organisational Change Questionnaire  [64] | - | - | 35-40 minutes to complete | - |
| NURSING HOMES | | | | |
| IPM  Intervention Process Measure  [67] | Effect size ranged from 0.11 - 0.26 | - | Missing items = 1.8% | - |
| SANN Scale  Staff Attitudes to Nutritional Nursing Care Scale  [66] | - | - | - | Culture/country – Sweden  Sample/setting – 232 geriatric nursing staff  Construct validity – 18 items, 5 factors  [105] |
| WHOLE COMMUNITIES/MULTIPLE SETTINGS | | | | |
| 4-E Telemeter  [70, 71] | - | - | - | - |
| Attitudes Towards Asthma Care Mobile Service Adoption Scale  [94] | Effect size ranged from 0.10 - 0.76 | - | - | - |
| Intention to Adopt Multimedia Messaging Service Scale  [69] | Effect size ranged from 0.05 - 0.64 | - | - | - |
| SOCIS  Systems of Care Implementation Survey  [68, 72] | - | - | - | - |
| SoCQ  Stages of Concern Questionnaire  [73, 74] | - | - | 10-15 minutes to complete | Culture/country – United States  Sample/setting – 142 professionals working with children with disabilities  Construct validity – 5 factors, 35 items; 5 factors, 15 items  [101]  Culture/country – Netherlands, Belgium  Sample/setting – 272 primary, secondary, and vocational teachers from 12 schools  Construct validity – 7 factors, 52 items  [112]  Culture/country – United States  Sample/setting – 376 algebra teachers attending inservice training at 16 sites  Construct validity – 5 factors, 27 items  [110]  Culture/country – Hong Kong  Sample/setting – 1,622 primary school teachers  Construct validity – 5 factors, 22 items  [104]  Culture/country – Turkey  Sample/setting – 1316 elementary school teachers and middle school maths teachers  Construct validity – 5 factors, 24 items  [106] |
| Telepsychotherapy Acceptance Questionnaire  [75] | Effect size ranged from 0.53 - 0.91 | - | Missing items = 1.7% | - |
| OTHER WORKPLACES/ORGANISATIONS | | | | |
| Adoption of Customer Relationship Management Technology Scale  [88] | - | - | - | - |
| Coping with Organisational Change Scale  [83] | - | - | - | - |
| DMRI  Data Mining Readiness Index  [80] | - | - | - | - |
| GII  Group Innovation Inventory  [78, 91] | - | - | - | Culture/country – Netherlands  Sample/setting – 261 home healthcare workers  Construct validity – 4 factors confirmed  [91] |
| Intention to Adopt Electronic Data Interchange Questionnaire  [79] | - | - | - | - |
| OCQ–C, P, R  Organisational Change Questionnaire – Climate of Change, Processes, and Readiness  [77] | - | - | - | Culture/country – England  Sample/setting – 799 individuals from a public sector agency  Construct validity – 41 items, 11 factors confirmed  [77] |
| OLCS  Organisational Learning Capacity Scale  [76] | - | - | - | - |
| Organisational Capacity Measure – Chronic Disease Prevention and Healthy Lifestyle Promotion  [81] | - | - | 43 minutes to complete | - |
| Organisational Environment and Processes Scale  [89] | - | - | - | - |
| PCI Scale  Perceived Characteristics of Innovating Scale  [87] | - | - | - | Culture/country – Cambodia  Sample/setting – 379 teacher-trainers  [109] |
| Perceived Strategic Value and Adoption of eCommerce Scale  [90] | *-* | - | 70 minutes to complete | - |
| PERM Questionnaire  Perceived eReadiness Model Questionnaire  [85, 86] | Effect size ≥ 0.8 | - | - | Culture/country – China  Sample/setting – 134 Managing directors of companies  [111]  Culture/country – Saudi Arabia  Sample/setting – 92 Managing directors, CEOs, other managers  [98] |
| Readiness for Organisational Change Measure  [82] | - | - | - | - |
| TAM2 Scale  Technology Acceptance Model 2 Scale  [96]  *Please Note:* The TAM 2 Scale has been cited over 9,000 times and adapted a number of times, the reporting of which is beyond the scope of this review. | - | - | - | Culture/country – Saudi Arabia  Sample/setting – 59 university employees  Construct validity – 5 factors, 20 items  [100]  Culture/country – Norway  Sample/setting – 186 employees from various organisations  Contruct validity – 8 factors, 23 items  [107] |
| TQM and Culture Survey  Total Quality Management and Culture Survey  [92] | - | - | - | - |
| WHPCI  Worksite Health Promotion Capacity Instrument  [84] | - | - | Missing items < 5% | - |

**Standard 13.3* – When indicators of effectiveness in program evaluations or policy studies are used, describe the method of constructing the indicator and report any technical problems (e.g. floor and ceiling effects, differences in variability across different measures).

***Standard 4.2 –* Test specifications should describe intended users, content of the test, test length, item formats, as well as psychometric properties.

****Standard 7.6* – For tests available in more than one language, describe procedures used to adapt/translate the test and details of reliability/validity.
