# Supplementary material for: Psychometric properties of implementation measures for public health and community settings and mapping of constructs against the Consolidated Framework for Implementation Research: a systematic review
Source: Implement Sci. 2016 Nov 8;11:148. doi: 10.1186/s13012-016-0512-5 (PMC5100177; doi:10.1186/s13012-016-0512-5)
Supplement: Additional file 6: — Mapping of measure domains against the 37 CFIR constructs [38–45, 47–92, 94, 96]. (DOCX 60 kb) [file 13012_2016_512_MOESM6_ESM.docx]

**Additional file 6: Table S1.** Mapping of measure domains against the 37 CFIR constructs.

|  |  | **Intervention Characteristics** | | | | | | | |  | **Outer Setting** | | | |  | **Inner Setting** | | | | | | | | | | | | | |  | **Characteristics**  **of Individuals** | | | | | |  | **Process** | | | | | | | |
| --- | --- | --- | --- | --- | --- | --- | --- | --- | --- | --- | --- | --- | --- | --- | --- | --- | --- | --- | --- | --- | --- | --- | --- | --- | --- | --- | --- | --- | --- | --- | --- | --- | --- | --- | --- | --- | --- | --- | --- | --- | --- | --- | --- | --- | --- |
|  |  | Intervention Source | Evidence Strength & Quality | Relative Advantage | Adaptability | Trialability | Complexity | Design Quality & Packaging | Cost |  | Client Needs & Resources | Cosmopolitanism | Peer Pressure | External Policy & Incentives |  | Structural Characteristics | Networks & Communications | Culture | Implementation Climate | Tension for Change | Compatibility | Relative Priority | Incentives & Rewards | Goals and Feedback | Learning Climate | Readiness for Implementation | Leadership Engagement | Available Resources | Access to Knowledge/Information |  | Knowledge & Beliefs | | Self-efficacy | Individual Stage of Change | Identification with Organisation | Other Personal Attributes |  | Planning | Engaging | Opinion Leaders | Internal Implementation Leaders | Champions | External Change Agents | Executing | Reflecting & Evaluating |
| **SCHOOLS** | | | | | | | | | | | | | | | | | | | | | | | | | | | | | | | | | | | | | | | | | | | | | |
| **Adopter Characteristics Scale**  [43] |  |  |  |  |  |  |  |  |  |  |  |  |  |  |  |  |  |  |  |  |  |  |  |  |  |  |  |  |  |  |  | |  |  |  | ✓ |  |  |  |  |  |  |  |  |  |
| **Awareness and Concern Instrument**  [51] |  |  |  |  |  |  |  |  |  |  |  |  |  |  |  |  |  |  |  |  |  |  |  |  |  |  |  |  |  |  | ✓ | |  |  |  |  |  |  |  |  |  |  |  |  |  |
| **HTSE Scale**  Health Teaching Self-efficacy Scale  [47] |  |  |  |  |  |  |  |  |  |  |  |  |  |  |  |  |  |  |  |  |  |  |  |  |  |  |  |  |  |  |  | | ✓ |  |  |  |  |  |  |  |  |  |  |  |  |
| **IITC-ESMH**  Index of Inter-professional Team Collaboration – Expanded School Mental Health  [50] |  |  |  |  |  |  |  |  |  |  |  |  |  |  |  |  | ✓ |  | ✓ |  |  |  |  |  |  |  |  |  |  |  |  | |  |  |  | ✓ |  |  |  |  |  |  |  |  |  |
| **MVAIS**  McKinney-Vento Act Implementation Scale  [40] |  |  |  |  |  |  |  |  |  |  |  | ✓ |  | ✓ |  |  |  |  |  |  |  |  |  |  |  |  |  | ✓ |  |  |  | |  |  |  |  |  |  |  |  |  |  |  | ✓ | ✓ |
| **Organisational Climate Instrument**  [51] |  |  |  |  |  |  |  |  |  |  |  |  |  |  |  |  |  | ✓ |  |  |  |  |  |  |  |  |  |  |  |  |  | |  |  | ✓ |  |  |  |  |  |  |  |  |  |  |
| **Perceived Attributes of the Healthy Schools Approach Scale**  [42] |  |  |  | ✓ |  |  | ✓ |  |  |  |  |  |  |  |  |  |  |  |  |  | ✓ |  |  |  |  |  |  |  |  |  |  | |  |  |  |  |  |  |  |  |  |  |  |  |  |
| **Policy Characteristics Scale**  [52] |  |  |  | ✓ |  |  |  |  |  |  |  |  |  |  |  |  |  |  |  |  |  |  |  |  |  |  |  | ✓ |  |  | ✓ | |  |  |  |  |  |  |  |  |  |  |  |  |  |
| **REBI**  Role-Efficacy Belief Instrument  [45] |  |  |  |  |  |  |  |  |  |  |  |  |  |  |  |  |  |  |  |  |  |  |  |  |  |  |  |  |  |  |  | | ✓ |  |  |  |  |  |  |  |  |  |  |  |  |
| **Rogers’s Adoption Questionnaire**  [51] |  |  |  | ✓ |  |  | ✓ |  |  |  |  |  |  |  |  |  |  |  |  |  |  |  |  |  |  |  |  |  |  |  |  | |  |  |  |  |  |  |  |  |  |  |  |  | ✓ |
| **School WPI**  School Wellness Policy Instrument  [48] |  |  |  |  |  |  |  |  |  |  |  |  |  | ✓ |  |  |  |  | ✓ |  |  |  |  | ✓ | ✓ |  |  | ✓ |  |  |  | | ✓ |  |  |  |  |  |  |  |  |  |  |  | ✓ |
| **SLEQ-SA**  School-level Environment Questionnaire – South Africa  [38] |  |  |  |  |  |  |  |  |  |  |  |  |  |  |  |  | ✓ | ✓ | ✓ |  |  |  |  |  | ✓ |  |  | ✓ |  |  |  | | ✓ |  | ✓ |  |  |  |  |  |  |  |  |  |  |
| **SSP-LO Measure**  School Success Profile – Learning Organisation Measure  [39] |  |  |  |  |  |  |  |  |  |  |  |  |  |  |  |  |  |  |  |  |  |  |  |  | ✓ |  |  |  |  |  |  | |  |  |  |  |  |  |  |  |  |  |  |  |  |
| **SRR-LQ**  School Readiness for Reforms – Leader Questionnaire  [41] |  |  |  |  |  |  |  |  |  |  |  |  |  |  |  |  |  |  | ✓ |  |  |  |  |  |  |  |  | ✓ |  |  |  | |  |  |  |  |  |  |  |  |  |  |  | ✓ | ✓ |
| **SUBSIST**  School-wide Universal Behaviour Sustainability Index – School Teams  [49] |  |  |  |  |  |  |  |  |  |  |  |  |  |  |  |  |  |  |  |  |  | ✓ |  | ✓ |  |  |  |  |  |  |  | |  |  |  |  |  |  |  |  | ✓ |  | ✓ | ✓ | ✓ |
| **Teacher Receptivity Measure**  [44] |  |  | ✓ |  |  |  |  |  |  |  |  |  |  |  |  |  |  |  | ✓ |  |  |  |  |  |  |  |  |  |  |  | ✓ | | ✓ |  |  | ✓ |  |  |  |  |  |  |  |  |  |
| **UNIVERSITIES/COLLEGES** | | | | | | | | | | | | | | | | | | | | | | | | | | | | | | | | | | | | | | | | | | | | | |
| **Intention to Adopt Mobile Commerce Questionnaire**  [54, 55] |  |  |  | ✓ |  | ✓ | ✓ |  |  |  |  |  | ✓ |  |  |  |  |  |  |  | ✓ |  |  |  |  |  |  |  |  |  | ✓ | |  | ✓ |  |  |  |  |  |  |  |  |  |  |  |
| **Perceived Attributes of eHealth Innovations Questionnaire**  [53] |  |  |  | ✓ |  | ✓ | ✓ |  |  |  |  |  |  |  |  |  |  |  |  |  | ✓ |  |  |  |  |  |  |  |  |  |  | |  |  |  |  |  |  |  |  |  |  |  |  |  |
| **Perceived Usefulness and Ease of Use Scale**  [56] |  |  |  | ✓ |  |  | ✓ |  |  |  |  |  |  |  |  |  |  |  |  |  |  |  |  |  |  |  |  |  |  |  |  | |  |  |  |  |  |  |  |  |  |  |  |  |  |
| **Post-adoption Information Systems Usage Measure**  [59] |  |  |  | ✓ |  |  | ✓ |  |  |  |  |  |  |  |  |  |  |  |  |  |  |  |  |  |  |  |  |  |  |  |  | |  |  |  | ✓ |  |  |  |  |  |  |  |  |  |
| **Social Influence on Innovation Adoption Scale**  [60] |  |  |  |  |  |  |  |  |  |  |  |  | ✓ |  |  |  | ✓ |  |  |  |  |  |  |  |  |  |  |  |  |  | ✓ | |  |  |  |  |  |  |  |  |  |  |  | ✓ |  |
| **TSROL**  Tertiary Students Readiness for Online Learning Scale  [57, 58] |  |  |  |  |  |  |  |  |  |  |  |  |  |  |  |  |  |  |  |  |  |  |  |  |  |  |  |  |  |  | ✓ | | ✓ |  |  | ✓ |  |  |  |  |  |  |  |  |  |
| **PHARMACIES** | | | | | | | | | | | | | | | | | | | | | | | | | | | | | | | | | | | | | | | | | | | | | |
| **Facilitators of Practice Change Scale**  [63] |  |  |  |  |  |  |  |  |  |  |  | ✓ |  | ✓ |  |  | ✓ |  |  |  |  |  |  |  |  |  |  | ✓ |  |  |  | |  |  |  |  |  |  |  |  |  |  | ✓ |  |  |
| **LATCon**  Leeds Attitude Towards Concordance Scale (Pharamacists)  [62] |  |  |  |  |  |  |  |  |  |  | ✓ |  |  |  |  |  |  |  |  |  |  |  |  |  |  |  |  |  |  |  | ✓ | |  |  |  |  |  |  |  |  |  |  |  |  |  |
| **Perceived Barriers to the Provision of Pharmaceutical Care Questionnaire**  [61] |  |  |  |  |  |  |  |  |  |  |  |  |  | ✓ |  |  |  |  |  |  |  |  |  |  |  |  |  | ✓ |  |  |  | |  |  |  |  |  |  |  |  |  |  |  |  |  |
| **POLICE/CORRECTIONAL FACILITIES** | | | | | | | | | | | | | | | | | | | | | | | | | | | | | | | | | | | | | | | | | | | | | |
| **Perceptions of Organisational Readiness for Change**  [65] |  |  |  |  |  |  |  |  |  |  |  | ✓ |  |  |  |  | ✓ |  | ✓ |  |  |  |  |  | ✓ |  |  | ✓ |  |  |  | | ✓ |  | ✓ |  |  |  |  |  |  |  |  | ✓ |  |
| **Receptivity to Organisational Change Questionnaire**  [64] |  |  |  |  |  |  |  |  |  |  |  |  |  |  |  |  |  |  |  |  |  |  |  |  |  | ✓ |  | ✓ |  |  | ✓ | |  |  | ✓ |  |  | ✓ |  |  |  |  |  |  |  |
| **NURSING HOMES** | | | | | | | | | | | | | | | | | | | | | | | | | | | | | | | | | | | | | | | | | | | | | |
| **IPM**  Intervention Process Measure  [67] |  |  |  | ✓ |  |  |  |  |  |  |  |  |  |  |  |  |  |  |  |  |  |  |  |  | ✓ |  | ✓ |  |  |  | ✓ | |  |  |  |  |  |  |  |  |  |  |  |  |  |
| **SANN Scale**  Staff Attitudes to Nutritional Nursing Care Scale  [66] |  |  |  |  |  |  |  |  |  |  | ✓ |  |  |  |  |  |  |  |  |  |  |  |  |  |  |  |  |  |  |  | ✓ | | ✓ |  |  |  |  |  |  |  |  |  |  |  |  |
| **WHOLE COMMUNITIES/MULTIPLE SETTINGS** | | | | | | | | | | | | | | | | | | | | | | | | | | | | | | | | | | | | | | | | | | | | | |
| **4-E Telemeter**  [70, 71] |  |  |  | ✓ |  |  |  |  |  |  |  |  |  |  |  |  |  |  |  |  |  |  | ✓ |  |  | ✓ |  |  | ✓ |  | |  |  |  |  | ✓ |  |  |  |  |  |  |  |  |  |
| **Attitudes Towards Asthma Care Mobile Service Adoption Scale**  [94] |  |  |  | ✓ |  |  | ✓ |  |  |  |  |  | ✓ |  |  |  |  |  |  |  |  |  |  |  |  |  |  |  |  |  | | ✓ |  |  |  | ✓ |  |  |  |  |  |  |  |  |  |
| **Intention to Adopt Multimedia Messaging Service Scale**  [69] |  |  |  | ✓ |  |  | ✓ | ✓ |  |  |  |  |  |  |  |  |  |  |  |  |  |  |  |  |  |  |  |  |  |  | |  |  |  |  | ✓ |  |  |  |  |  |  |  |  |  |
| **SOCIS**  Systems of Care Implementation Survey  [68, 72] |  |  |  |  | ✓ |  |  |  |  |  | ✓ | ✓ |  |  |  |  |  |  |  |  |  |  |  |  | ✓ |  |  | ✓ |  |  | |  |  |  |  |  |  | ✓ |  |  |  |  |  |  | ✓ |
| **SoCQ**  Stages of Concern Questionnaire  [73, 74] |  |  |  |  |  |  |  |  |  |  | ✓ |  |  |  |  |  |  |  | ✓ |  |  |  |  |  |  |  |  | ✓ |  |  | | ✓ |  |  |  |  |  |  |  |  |  |  |  |  | ✓ |
| **Telepsycho-therapy Acceptance Questionnaire**  [75] |  |  |  | ✓ |  |  | ✓ |  |  |  |  |  |  |  |  |  |  |  |  |  |  |  |  |  |  |  |  |  |  |  | | ✓ |  |  |  | ✓ |  |  |  |  |  |  |  |  |  |
| **OTHER WORKPLACES/ORGANISATIONS** | | | | | | | | | | | | | | | | | | | | | | | | | | | | | | | | | | | | | | | | | | | | | |
| **Adoption of Customer Relationship Management Technology Scale**  [88] |  |  |  | ✓ |  |  |  |  | ✓ |  |  |  | ✓ |  |  |  |  |  |  |  |  |  |  |  |  |  |  |  | ✓ |  |  | |  |  |  | ✓ |  |  |  |  |  |  |  |  |  |
| **Coping with Organisational Change Scale**  [83] |  |  |  |  |  |  |  |  |  |  |  |  |  |  |  |  |  |  |  |  |  |  |  |  |  |  |  |  |  |  |  | |  |  |  | ✓ |  |  |  |  |  |  |  |  |  |
| **DMRI**  Data Mining Readiness Index  [80] |  |  |  |  |  |  |  |  |  |  |  |  |  |  |  |  |  | ✓ |  |  |  |  |  |  |  |  |  | ✓ | ✓ |  |  | |  |  |  |  |  |  |  |  | ✓ |  |  |  |  |
| **GII**  Group Innovation Inventory  [78, 91] |  |  |  |  |  |  |  |  |  |  |  |  |  |  |  |  |  |  | ✓ |  |  |  |  |  | ✓ |  |  |  |  |  |  | |  |  |  |  |  |  |  |  |  |  |  |  |  |
| **Intention to Adopt Electronic Data Interchange Questionnaire**  [79] |  |  |  | ✓ |  |  |  |  |  |  |  |  | ✓ |  |  |  |  |  |  |  |  |  |  |  |  |  |  | ✓ |  |  |  | |  |  |  |  |  |  |  |  |  |  |  |  |  |
| **OCQ–C, P, R**  Organisational Change Questionnaire – Climate of Change, Processes, and Readiness  [77] |  |  |  |  |  |  |  |  |  |  |  |  |  |  |  | ✓ | ✓ | ✓ | ✓ |  |  |  |  |  | ✓ |  | ✓ |  |  |  | ✓ | |  |  |  | ✓ |  |  |  |  |  |  |  |  |  |
| **OLCS**  Organisational Learning Capacity Scale  [76] |  |  |  |  |  |  |  |  |  |  |  |  |  |  |  |  | ✓ | ✓ |  |  |  |  |  |  | ✓ |  |  |  |  |  |  | |  |  |  |  |  |  |  |  |  |  |  |  |  |
| **Organisational Capacity Measure – Chronic Disease Prevention and Healthy Lifestyle Promotion**  [81] |  |  |  |  |  |  |  |  |  |  |  | ✓ |  |  |  | ✓ |  |  |  |  |  |  |  |  |  |  | ✓ | ✓ | ✓ |  |  | |  |  |  |  |  | ✓ |  |  |  |  |  |  | ✓ |
| **Organisational Environment and Processes Scale**  [89] |  |  |  |  |  |  |  |  |  |  |  |  |  |  |  |  |  |  | ✓ |  |  | ✓ | ✓ |  |  | ✓ |  | ✓ |  |  |  | |  |  |  |  |  |  |  |  |  |  |  |  |  |
| **PCI Scale**  Perceived Characteristics of Innovating Scale  [87] |  |  |  | ✓ |  | ✓ | ✓ |  |  |  |  |  |  |  |  |  |  |  |  |  | ✓ |  |  |  |  |  |  |  |  |  |  | |  |  |  |  |  |  |  |  |  |  |  |  |  |
| **Perceived Strategic Value and Adoption of eCommerce Scale**  [90] |  |  |  | ✓ |  |  | ✓ |  |  |  |  |  | ✓ |  |  |  |  |  |  |  | ✓ |  |  |  |  |  |  |  |  |  |  | | ✓ |  |  |  |  |  |  |  |  |  |  |  |  |
| **PERM Questionnaire**  Perceived eReadiness Model Questionnaire  [85, 86] |  |  |  | ✓ |  |  |  |  |  |  |  |  | ✓ | ✓ |  |  |  |  |  |  |  |  |  |  |  | ✓ | ✓ | ✓ |  |  |  | |  |  |  |  |  |  |  |  |  |  |  |  |  |
| **Readiness for Organisational Change Measure**  [82] |  |  |  | ✓ |  |  |  |  |  |  |  |  |  |  |  |  |  |  |  |  |  |  |  |  |  |  | ✓ |  |  |  | ✓ | | ✓ |  |  |  |  |  |  |  |  |  |  |  |  |
| **TAM2 Scale**  Technology Acceptance Model 2 Scale  [96] |  |  |  | ✓ |  |  | ✓ |  |  |  |  |  |  |  |  |  |  |  | ✓ |  |  |  | ✓ |  |  |  |  |  |  |  | ✓ | |  |  |  |  |  |  |  |  |  |  |  |  | ✓ |
| **TQM and Culture Survey**  Total Quality Management and Culture Survey  [92] |  |  |  |  |  |  |  |  |  |  | ✓ |  |  |  |  |  | ✓ |  |  |  |  |  |  |  | ✓ |  | ✓ | ✓ |  |  |  | |  |  |  |  |  |  |  |  |  |  |  |  |  |
| **WHPCI**  Worksite Health Promotion Capacity Instrument  [84] |  |  |  |  |  |  |  |  |  |  |  |  |  |  |  |  |  |  | ✓ |  |  |  |  |  |  | ✓ |  |  |  |  |  | |  |  |  |  |  |  |  |  |  |  |  |  | ✓ |
| **TOTAL No. Measures Addressing Each Construct** |  | 0 | 1 | 19 | 1 | 3 | 12 | 1 | 1 |  | 5 | 5 | 7 | 5 |  | 2 | 8 | 5 | 12 | 0 | 5 | 2 | 3 | 2 | 10 | 5 | 6 | 17 | 4 |  | 16 | | 10 | 1 | 4 | 12 |  | 3 | 0 | 0 | 2 | 0 | 2 | 5 | 10 |
